# Flavonoid intake is associated with lower all-cause and disease-specific mortality: The National Health and Nutrition Examination Survey 2007–2010 and 2017–2018

**DOI:** 10.3389/fnut.2023.1046998

**Published:** 2023-02-14

**Authors:** Fengying Zhou, Ke Gu, Yanjun Zhou

**Affiliations:** ^1^Department of Breast Diseases, Wuxi Meternal and Child Health Hospital, Women’s Hospital of Jiangnan University, Jiangnan University, Wuxi, Jiangsu, China; ^2^Department of Radiotherapy and Oncology, Affiliated Hospital of Jiangnan University, Wuxi, Jiangsu, China

**Keywords:** diet, flavonoids, all-cause mortality, heart diseases, stroke

## Abstract

Adequate intake of flavonoids may influence mortality, particularly of heart and cerebrovascular diseases. However, the importance of each flavonoid and its subclasses in preventing all-cause and disease-specific mortalities remain unclear. In addition, it is unknown which population groups would benefit from high flavonoid intake. Therefore, personalized mortality risk based on flavonoid intake needs to be estimated. The association between flavonoid intake and mortality among 14,029 participants in the National Health and Nutrition Examination Survey was examined using Cox proportional hazards analysis. A prognostic risk score and nomogram linking flavonoid intake and mortality were constructed. During a median follow-up of 117 months (9.75 years), 1,603 incident deaths were confirmed. Total flavonol intake was associated with a significantly reduced all-cause mortality [multivariable adjusted hazard ratio [HR] (95% confidence interval [CI]), 0.87 (0.81, 0.94), *p* for trend <0.001], especially in participants aged 50 years and older and former smokers. Similarly, total anthocyanidin intake was inversely associated with all-cause mortality [0.91 (0.84, 0.99), *p* for trend = 0.03], which was most significant in non-alcoholics. The intake of isoflavones was negatively associated with all-cause mortality [0.81 (0.70, 0.94), *p* = 0.01]. Moreover, a risk score was constructed based on survival-related flavonoid intake. The constructed nomogram accurately predicted the all-cause mortality of individuals based on flavonoid intake. Taken together, our results can be used to improve personalized nutrition.

## 1. Introduction

Cardiovascular diseases, including fatal coronary events, myocardial infarction, ischemic stroke, and hemorrhagic stroke (1) are the most common causes of death worldwide (2, 3). Therefore, primary prevention of cardiovascular disease is a major public health priority. Effective primary prevention strategies targeting modifiable risk factors are essential for reducing cardiovascular disease burden in the population. A diet emphasizing fruits, vegetables, and cereal grains has been shown to lower the risk of cardiovascular disease-related mortality (4).

Adequate intake of fruits, vegetables, and cereal grains is recommended to reduce the risk of cardiovascular disease and all-cause mortality (5). The benefits of plant-based diets may be partially attributed to flavonoids. Flavonoids are present in fruits, vegetables, cereals, and beverages, including tea and red wine, underlining the benefits of sufficient intake of these products (6). Flavonoids are water-soluble polyphenolic molecules (7). Flavones, anthocyanidins, flavanones, flavonols, flavan-3-ols, and isoflavones are the most common flavonoids in the human diet (8). Many flavonoids have antioxidant, free-radical scavenging, and anti-inflammatory properties (9). However, the available data on the association between flavonoid intake and cardiovascular diseases and all-cause mortality are limited and inconsistent (10, 11, 12, 13, 14). Herein, the relationship between flavonoid intake and all-cause mortality, as well as disease-specific mortality, was analyzed using the National Health and Nutrition Examination Survey (NHANES) data collected from 2007 to 2010 and 2017 to 2018. In addition, this study examined whether participants with unhealthy lifestyle behaviors could benefit from a high flavonoid intake.

## 2. Materials and methods

### 2.1. National Health and nutrition examination survey (NHANES)

The NHANES is a cross-sectional survey using a stratified, multistage probability sampling design. The protocol of the NHANES was approved by the Research Ethics Review Board of the National Centre for Health Statistics, and all participants signed written informed consent. For this survey, all data are publicly available and without personal identifiable information, and all methods were carried out in accordance with relevant guidelines and regulations. The first interview on demographic and health-related information was conducted in the participants’ homes. Approximately 2 weeks later, a standardized physical examination was conducted in a mobile examination center, as well as a blood draw, 24-h dietary recall and other investigations. For this study, we included 18,538 adults aged 18 years or older who participated in the continuous NHANES cycles of 2007–2010 and 2017–2018; with available mortality information, 14,490 adults participated in the dietary flavonoid intake assessment. The characteristics of participants without the information on dietary flavonoid intake were summarized in the Supplementary Table S1. All analyses used weighted samples (“wtdr2d”) and considered the stratification and clustering of the design to derive estimates that were applicable to the United States (US) population (15).

### 2.2. Dietary flavonoid intake assessment

Intakes of total flavonoids and each flavonoid subclass in our study were downloaded from Flavonoid Values for US Department of Agriculture Survey Foods and Beverages (16), which are derived from (i) Database of Flavonoid Values for Food Codes; (ii) Flavonoid Intake Data Files from What We Eat in America and NHANES. The flavonoid database included data on the 6 main flavonoid subclasses: (i) flavones (apigenin and luteolin); (ii) anthocyanins (cyanidin, delphinidin, malvidin, pelargonidin, peonidin, and petunidin); (iii) flavanones (eriodictyol, hesperetin, and naringenin), (iv) flavonols (isorhamnetin, kaempferol, myricetin, and quercetin); (v) flavan-3-ols [catechins including: (−)-epicatechin, (−)-epicatechin 3-gallate, (−)-epigallocatechin, (−)-epigallocatechin 3-gallate, (+)-catechin, (+)-gallocatechin; theaflavin, theaflavin-3,3′-digallate, theaflavin-3′-gallate, theaflavin-3-gallate, and thearubigins]; (vi) isoflavones (daidzein, genistein, and glycitein), total flavones, total anthocyanidins, total flavanones, total flavan-3-ols, total flavonols, total isoflavones, subtotal catechins, and total flavonoids Daily flavonoid intake per participant was determined on the first and second days, and the mean of the two-day flavonoid intake was used in subsequent analyses.

### 2.3. Urinary phytoestrogens assessment

The levels of isoflavone metabolites, daidzein (ng/mL), equol (ng/mL), genistein (ng/mL), and O-desmethylangolensin (ODMA, ng/mL) as well as lignan metabolites, enterodiol (ng/mL) and enterolactone (ng/mL) in the urine were measured by high-performance liquid chromatography-atmospheric pressure photoionization-tandem mass spectrometry. The association between urinary phytoestrogen levels and isoflavone intake in the years 2007–2010 was analyzed using the Pearson correlation method.

### 2.4. Mortality ascertainment

The follow-up period was updated until December 31, 2019. We used the time in months from the household interview until death or lost to follow-up as the follow-up duration. The outcome was the mortality status ascertained using the National Death Index. The cause of death was defined according to the International Classification of Diseases (ICD) 10. The relationship between quartile of flavonoid intake and all-cause mortality and specific disease mortality was determined using Cox proportional hazards analysis. The association between mortality and flavonoid intake was evaluated by univariate and multivariate Cox analysis with an increment unit of 10 mg/day. Subsequent Cox models were stratified according to age, sex, PIR, race, smoking status, and drinking status with the increment unit of 10 mg/day.

### 2.5. Covariates assessment

Information on age, race, education, marital status, poverty income ratio (PIR), smoking status, alcohol use, and physical activity (PA) was collected using questionnaires. In terms of education status, less than 9th grade was coded as 1; 9–11th grade (includes 12th grade with no diploma) as 2, high school graduation or equivalent as 3, some college or associates degree as 4, and college graduate or above as 5. For marital status, married was coded as 1, divorced as 2, separated as 3, never married as 4, widowed as 5, and living with partner as 6. Participants were categorized based on PIR as follows: participants with a PIR low than the 30th percentile of the PIR as “PIR low” (as reference); those with a PIR greater than or equal to the 60th percentile of the PIR as “PIR high,” and the remaining participants as “PIR medium.” Body mass index (BMI) was calculated as weight (kg)/height^2^ (m^2^). The term “never” with smoking use was defined as less than 100 cigarettes during their lifetime; “former” as more than 100 cigarettes during their lifetime but not currently smoking; and “now” as more than 100 cigarettes during their lifetime and currently smoking some days or every day. The classification of alcohol usage was as previously described (17). The healthy eating index (HEI) was calculated for each participant based on the 2015 version, using the sum of the HEI of the first and second days (18). The dietary inflammatory index (DII) was calculated as previously described (19). PA was calculated using the weekly total time of activity and total metabolic equivalent (MET).

Hyperlipidemia was defined as triglycerides ≥150 mg/dL, low-density lipoprotein ≥130 mg/dl, high-density lipoprotein <140 ng/dL, or the use of lipid-lowering drugs. Cardiovascular disease was defined as a history of a heart attack or stroke. The participants were diagnosed as chronic obstructive pulmonary disease (COPD) when they met one of the following conditions: the value of forced expiratory volume at first second/forced vital capacity (FEV1/FVC) < 0.7 after usage of a bronchodilator; reported emphysema; use COPD drugs, selective phosphodiesterase-4 inhibitors, mast cell stabilizers leukotriene modifiers, and inhaled corticosteroids. Participants were diagnosed with asthma when they met one of the following conditions: asthma, asthma attack, application of selective phosphodiesterase-4 inhibitors, mast cell stabilizers, leukotriene modifiers, and inhaled corticosteroids. Participants with both COPD and asthma were defined as those with ACO. Stroke history was defined as having had a stroke. A cancer history was defined as the presence of cancer at any point. The average blood pressure was calculated as previously described (20) and participants met one of the following conditions: previously told to have hypertension; taking anti-hypertensive drugs; or with systolic pressure ≥ 140 mmHg or diastolic pressure ≥ 90 mmHg were diagnosed with hypertension. Participants were diagnosed as type 2 diabetes mellitus (DM) when they met one of the following conditions: clinical diagnosis of diabetes; HbA1c ≥ 6.5%; fasting glucose ≥7.0 mmol/l; glucose ≥11.1 mmol/l; oral glucose tolerance test ≥11.1 mmol/l; usage of antidiabetic drugs. In addition, missing values in education status, marital status, PIR, BMI and total time/MET of PA were imputed by the random forest method in the R package “mice.”

### 2.6. Construction and validation of risk score

The cohort was randomly subdivided into training and testing datasets at a ratio of 4:1. The risk score was exported for each participant using the following equation:


$$\text{Risk Score}=\overset{\infty }{\underset{n=1}{\sum }}\left(\text{intake level}*\mathrm{Coefficient}\right)$$

The intake level is the intake level of each flavonoid and the coefficient represents the corresponding univariate Cox proportional hazard regression coefficient. The association of the risk score with all-cause mortality and disease-specific mortality was assessed using Cox proportional hazards analysis.

### 2.7. Statistical analysis

Continuous variables are presented as mean ± standard deviation, or mean (95% confidence intervals, 95% CIs) as indicated below each table, and categorical variables are presented as percentage (95% CIs). Cox proportional hazard models were used to calculate hazard ratios (HRs) and 95% CIs. The time in the Cox proportional model, as follow-up duration, was recorded from the household interview until death or lost to follow-up in months. The association among flavonoid intake and the association between isoflavone intake and urinary phytoestrogens were evaluated by the function “cor” and the R package “corrplot,” in which, *p* value <0.0001 was defined as significant and the absolute value of Pearson’s correlation coefficient *r* ≥ 0.8 was classified as strong correlation, 0.8 < *r* ≤ 0.5 as moderate correlation and 0.5 < *r* ≤ 0.2 as weak correlation; *r* < 0.2 as no correlation. In the remaining analysis, a *p* value < 0.05 was used as a cut-off for statistical significance. A feasible nomogram for weighted survey data was established and validated by calibration curve for predicting the 12.5-year survival probability in participants using the “rms” and “SvyNom” package in R (21). All analyses were conducted using the R software (version 4.1.3, the R Foundation for Statistical Computing, 181 Longwood Ave, Boston, MA 02115). The R package “nhanseR” and “survey” were employed for the data preparation and statistics analysis.

## 3. Results

### 3.1. Baseline characteristics

The 14,029 NHANES participants with valid information on flavonoid intake represented 227.1 million noninstitutionalized residents of the United States in the years 2007–2010 and 2017–2018. The final study population had a mean age of 50.29 years and 47.72% were males. The median follow-up duration was 117 months (9.75 years, range, 1–160 months). A total of 1,603 (12.5%) participants died during the follow-up period until December 31^st^, 2019. The characteristics of the deaths are summarized in Supplementary Table S2. Compared to those who were alive, participants who had died were older (66.85 ± 0.57, *p* < 0.0001), male (51.57%, *p* = 0.02), white (*p* < 0.0001), undereducated (*p* < 0.0001), current smokers (*p* < 0.0001), with lower PIR (2.49 ± 0.08, *p* < 0.0001), with higher DII (1.80 ± 0.07, *p* < 0.0001), and with less total time and total MET of PA (884.01 ± 76.54, 3536.04 ± 306.17, *p* < 0.0001) (Supplementary Table S2). In addition, participants who died had a higher prevalence of hyperlipidemia, diabetes, hypertension, cardiovascular diseases, cancer, or respiratory diseases. There were significant differences in flavonoid intake between participants who died and those who were still alive (Supplementary Table S2). The majority of those who died showed a low intake of total isoflavones, total flavones, total flavonols, daidzein, genistein, glycitein, delphinidin, peonidin, eriodictyol, apigenin, luteolin, isorhamnetin, kaempferol, myricetin, and quercetin, but a higher intake of total flavanones and hesperetin (Supplementary Table S2).

### 3.2. Flavonoid intake

Because of the strong association between total flavonol intake and death (Supplementary Table S2, *p* = 0.003), the relevant baseline variables were further analyzed according to the total flavonol intake quartiles (Table 1). As expected, with an increased intake of flavonols, the participants were older, with higher PIR, higher HEI score, lower DII, fewer current smokers, less heavy alcohol users, less ACO prevalence, and lower stroke incidence (Table 1).

**Table 1:** Relevant baseline variables according to the total flavonol intake group.

	Total intake of flavonols (mg/day)				*p* value
	1Q	2Q	3Q	4Q	
	<6.815	6.815–12.555	12.555–22.105	≥22.105	
**Baseline sociodemographic, lifestyle, and health-related variables**					
Age, years	45.37 ± 0.55	47.02 ± 0.53	47.39 ± 0.50	48.08 ± 0.49	<0.001
Sex, %					<0.0001
Female	59.89 (57.69, 62.09)	54.23 (52.26, 56.19)	52.30 (49.95, 54.66)	45.92 (43.64, 48.20)	
Male	40.11 (37.91, 42.31)	45.77 (43.81, 47.74)	47.70 (45.34, 50.05)	54.08 (51.80, 56.36)	
Race, %					<0.0001
White	63.97 (59.13, 68.81)	64.43 (59.97, 68.88)	66.48 (62.38, 70.59)	71.73 (67.88, 75.58)	
Black	15.31 (12.30, 18.32)	12.42 (10.02, 14.82)	10.66 (8.80, 12.52)	8.19 (6.86, 9.52)	
Mexican	9.41 (7.01, 11.81)	9.66 (7.11, 12.20)	9.48 (7.27, 11.70)	6.40 (4.58, 8.21)	
Other	11.31 (8.96, 13.66)	13.49 (11.37, 15.62)	13.37 (10.62, 16.12)	13.69 (11.31, 16.06)	
Education, %					<0.0001
1	6.89 (5.61, 8.16)	5.95 (4.85, 7.04)	4.84 (3.80, 5.89)	2.86 (2.30, 3.41)	
2	14.44 (12.72, 16.17)	10.64 (9.02, 12.27)	10.19 (8.53, 11.84)	8.10 (7.05, 9.15)	
3	29.21 (26.65, 31.77)	25.78 (23.47, 28.08)	23.04 (21.02, 25.07)	22.53 (20.06, 24.99)	
4	29.76 (27.03, 32.48)	31.88 (28.97, 34.79)	29.12 (26.49, 31.76)	30.03 (27.59, 32.48)	
5	19.70 (17.11, 22.29)	25.75 (22.02, 29.49)	32.80 (29.45, 36.16)	36.48 (32.75, 40.22)	
Marital status, %					<0.001
					
1	48.61 (45.58, 51.64)	54.88 (52.09, 57.68)	57.93 (54.40, 61.46)	57.97 (55.00, 60.94)	
2	11.26 (9.58, 12.94)	10.12 (8.69, 11.55)	8.19 (7.12, 9.25)	9.93 (8.37, 11.48)	
3	2.62 (1.88, 3.37)	2.90 (2.15, 3.65)	2.02 (1.47, 2.57)	2.42 (1.77, 3.06)	
4	21.83 (19.32, 24.34)	19.50 (17.45, 21.55)	18.08 (15.38, 20.77)	17.34 (15.54, 19.14)	
5	7.07 (5.88, 8.25)	5.84 (4.80, 6.87)	6.28 (5.07, 7.49)	4.26 (3.59, 4.93)	
6	8.61 (7.33, 9.89)	6.76 (5.53, 7.99)	7.51 (6.29, 8.72)	8.08 (5.82, 10.35)	
PIR	2.63 ± 0.06	2.87 ± 0.06	3.13 ± 0.05	3.32 ± 0.05	<0.0001
BMI (kg/m^2^)	29.57 ± 0.26	29.34 ± 0.17	29.05 ± 0.18	28.72 ± 0.21	0.02
Total score of HEI	46.53 ± 0.37	52.94 ± 0.36	55.23 ± 0.45	57.16 ± 0.56	<0.0001
DII	2.57 ± 0.05	1.71 ± 0.05	1.21 ± 0.06	0.65 ± 0.07	<0.0001
Total time of PA (mins/ week)	1366.40 ± 57.78	1205.75 ± 35.35	1224.12 ± 45.59	1350.02 ± 55.82	0.01
Total MET of PA (/week)	5,407.67 ± 263.51	4,872.56 ± 163.15	4,845.24 ± 217.90	5,337.79 ± 241.65	0.12
Smoking status, %					<0.001
Former	23.30 (20.91, 25.70)	22.93 (20.69, 25.18)	25.86 (22.95, 28.77)	25.32 (23.21, 27.42)	
Never	52.88 (49.97, 55.80)	58.84 (55.90, 61.78)	57.74 (54.56, 60.92)	56.55 (53.84, 59.26)	
Now	23.81 (20.93, 26.69)	18.22 (16.18,20.26)	16.40 (14.28, 18.53)	18.13 (16.18, 20.09)	
Alcohol usage, %					<0.0001
Former	14.04 (11.69, 16.38)	12.62 (10.76, 14.49)	9.45 (7.87, 11.04)	9.31 (8.04, 10.59)	
Heavy	22.30 (19.63, 24.97)	20.93 (18.30, 23.55)	21.93 (19.35, 24.51)	22.16 (19.85, 24.48)	
Mild	31.69 (28.13, 35.25)	38.20 (34.81, 41.58)	40.26 (37.06, 43.47)	41.44 (39.00, 43.89)	
Moderate	18.60 (16.60, 20.60)	16.07 (13.96, 18.19)	18.58 (16.20, 20.96)	18.80 (16.85, 20.76)	
Never	13.37 (11.75, 14.99)	12.18 (10.20, 14.16)	9.77 (8.12, 11.43)	8.28 (6.77, 9.78)	
**Disease diagnosis at interview**					
Hyperlipidemia, %					0.73
No	31.49 (28.68, 34.31)	31.08 (28.44, 33.72)	30.32 (27.11, 33.53)	32.25 (29.84, 34.65)	
Yes	68.51 (65.69, 71.32)	68.92 (66.28, 71.56)	69.68 (66.47, 72.89)	67.75 (65.35, 70.16)	
Cardiovascular diseases, %					0.03
No	89.95 (88.22, 91.67)	90.09 (88.98, 91.21)	91.59 (90.26, 92.92)	91.98 (90.87, 93.10)	
Yes	10.05 (8.33, 11.78)	9.91 (8.79, 11.02)	8.41 (7.08, 9.74)	8.02 (6.90, 9.13)	
Respiratory system disease, %					0.14
ACO	2.73 (2.05, 3.41)	2.23 (1.54, 2.91)	1.84 (1.13, 2.56)	2.00 (1.22, 2.78)	
Asthma	12.66 (10.77, 14.55)	12.00 (9.84, 14.15)	11.45 (10.22, 12.68)	10.64 (9.41, 11.87)	
COPD	2.23 (1.50, 2.96)	2.96 (2.31, 3.61)	3.96 (2.81, 5.12)	3.03 (2.22, 3.83)	
No	82.38 (80.17, 84.58)	82.82 (80.51, 85.13)	82.74 (80.53, 84.95)	84.34 (82.61, 86.06)	
Stroke, %					0.002
No	95.56 (94.61, 96.50)	96.09 (95.45, 96.73)	96.88 (96.08, 97.68)	97.63 (97.00, 98.26)	
Yes	4.44 (3.50, 5.39)	3.91 (3.27, 4.55)	3.12 (2.32, 3.92)	2.37 (1.74, 3.00)	
Cancer					0.68
No	90.54 (89.20, 91.89)	89.69 (88.43, 90.95)	89.69 (88.34, 91.04)	90.58 (88.86, 92.30)	
Yes	9.46 (8.11, 10.80)	10.31 (9.05, 11.57)	10.31 (8.96, 11.66)	9.42 (7.70, 11.14)	
Hypertension, %					0.2
No	61.34 (58.85, 63.82)	62.86 (59.64, 66.09)	65.20 (62.74, 67.65)	62.74 (60.14, 65.34)	
Yes	38.66 (36.18, 41.15)	37.14 (33.91, 40.36)	34.80 (32.35, 37.26)	37.26 (34.66, 39.86)	
DM, %					0.28
No	77.55 (75.43, 79.67)	78.62 (76.19, 81.05)	79.04 (76.88, 81.19)	80.28 (78.22, 82.35)	
Yes	22.45 (20.33, 24.57)	21.38 (18.95, 23.81)	20.96 (18.81, 23.12)	19.72 (17.65, 21.78)	

Continuous variables are presented as mean ± standard deviation and categorical variables are presented as percentage (95% confidence intervals, 95% CIs). PIR, poverty income ratio; BMI, body mass index; HEI, Healthy Eating Index 2015 version; DII, dietary inflammatory index; PA, physical activity; MET, metabolic equivalent; COPD, chronic obstructive pulmonary disease; DM, type 2 diabetes mellitus. In education, less than 9th grade was coded as 1; 9–11th grade (includes 12th grade with no diploma) as 2, high school graduation or equivalent as 3, some college or associates degree as 4, and college graduate or above as 5. For marital status, married was coded as 1, divorced as 2, separated as 3, never married as 4, widowed as 5, and living with partner as 6.

As shown in Table 1, there was a strong relationship between flavonol intake and age, race, and PIR (*p* < 0.001). Age-adjusted and race-adjusted mean intakes of flavonols across the PIR groups were compared using linear regression. Relative to the PIR low group, the female and male younger than 50 in the PIR high group had higher mean intake of flavonols (*p* < 0.001) (Table 2). The female aged 50 and above and the male younger than 50 in the PIR medium group had higher mean intake of flavonols, compared to the relevant participants in PIR low group (*p* < 0.05) (Table 2). The results may suggest that economic status may influence dietary intake of flavonols.

**Table 2:** Mean intake of total flavonols by age, sex, and PIR group.

Sex and age group	Participants (*n*)	Total Flavonols (mg/day)	Participants (*n*)	Total Flavonols (mg/day)	Participants (*n*)	Total Flavonols (mg/day)
	PIR-low		PIR-medium		PIR-high	
All	4,213	15.30 (14.46, 16.14)	4,221	17.19 (16.16, 18.22)	5,595	20.22 (19.35, 21.09)
Female < 50	1,356	13.84 (12.93, 14.74)	1,024	15.18 (13.73, 16.62)	1,270	17.69 (16.41, 18.96)***
Male < 50	986	16.10 (14.73, 17.46)	940	18.52 (16.91, 20.13)*	1,204	21.14 (19.49, 22.79)***
Female ≥ 50	1,021	14.74 (13.58, 15.90)	1,154	17.21 (15.76, 18.66)**	1,507	20.51 (18.83, 22.19)***
Male ≥ 50	850	18.59 (15.73, 21.45)	1,103	18.14 (16.32, 19.96)	1,614	21.67 (20.21, 23.14)

Age-adjusted and race-adjusted mean intake of flavonols across the PIR groups were compared using linear regression. The intake of flavonols are presented as means (95%CIs). *p < 0.05, **p < 0.01, ***p < 0.001; PIR, poverty income ratio.

**Figure 1:**
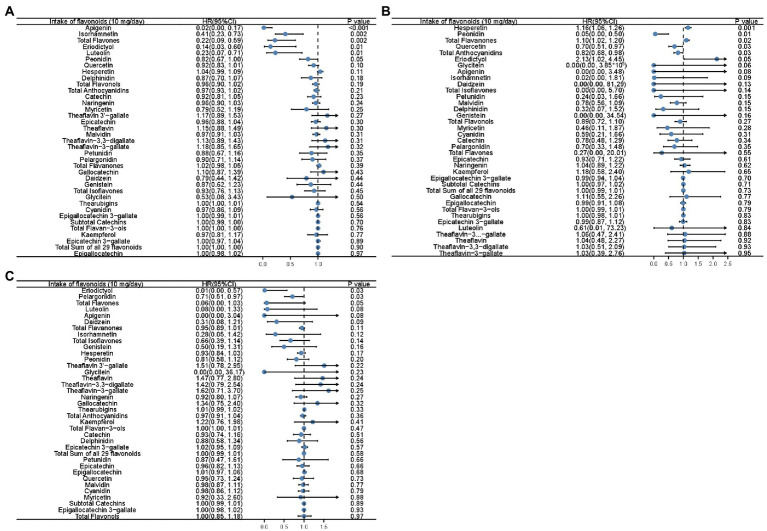
Forest plots showing the association between flavonoid intake and all-cause mortality **(A)**, mortality of heart diseases **(B)**, and mortality of cerebrovascular diseases **(C)** in Cox analysis adjusted for age and PIR with the increment unit of 10 mg/day. HR: Hazard ratios; PIR: poverty income ratio.

The association between mortality and flavonoid intake was further explored considering an increment unit of 10 mg/day (Supplementary Figure S1; Figure 1). Unadjusted Cox analysis revealed that all-cause mortality was inversely associated with the intake of total flavones, total flavonols, eriodictyol, luteolin, apigenin, isorhamnetin, quercetin, and kaempferol (Supplementary Figure S1). Simultaneously, there was a positive association between the intake of total flavanones and hesperetin and all-cause mortality (Supplementary Figure S1A). Moreover, all-cause mortality was inversely related to the intake of total flavones, apigenin, luteolin, isorhamnetin, peonidin, and eriodictyol after adjusting for PIR and age (Figure 1A). Moreover, unadjusted Cox analysis revealed an inverse association between heart disease mortality and the intake of total flavones, eriodictyol, luteolin, isorhamnetin, and apigenin (Supplementary Figure S1B). After adjusting for PIR and age, it was found that the intake of total anthocyanidins, peonidin, and quercetin was inversely associated with the risk of death from heart disease (Figure 1B). Subsequently, the mortality from cerebrovascular diseases was inversely associated with the intake of total flavonols, quercetin, peonidin, and glycitein in the unadjusted Cox analysis (Supplementary Figure S1C), and the intake of eriodictyol and pelargonidin remained inversely associated with the mortality from cerebrovascular diseases after adjustment for PIR and age (Figure 1C). However, the intake of some flavonoids, including total flavanones, hesperetin, and naringenin, was associated with an increased risk of death from cerebrovascular diseases in the unadjusted Cox analysis (Supplementary Figure S1C). Total flavanones, hesperetin, and eriodictyol remained risk factors for heart disease death, even after adjustment for PIR and age (Figure 1B).

Cox analysis was performed to identify whether the intake of each subclass was independently associated with mortality (Table 3). Intake of anthocyanidins was inversely associated with all-cause mortality (HR [95%CI], 0.91 [0.84, 0.99], *p* for trend = 0.03) (Table 3). Flavonol intake was inversely associated with all-cause mortality (0.87 [0.81, 0.94], *p* for trend <0.001, Table 3). There was a trend that the intake of flavonols was inversely associated with cerebrovascular disease mortality (0.81 [0.64, 1.01], *p* for trend = 0.06) (Table 3). The intake of flavan-3-ol was tended to be inversely associated with reduced cerebrovascular mortality (0.81 [0.65, 1.01], *p* = 0.06).

**Table 3:** Hazard ratios of mortality by quartiles of flavonoid intake.

	Q1	Q2	Q3	Q4	HR (95%CI)	*p* for trend
Total flavonoids (mg/day)	<24.33	24.33–64.13	64.13–217.64	≥ 217.64		
Case number (*n*)	3,508	3,507	3,507	3,507		
Person-years	26,243	27,338	26,263	27,922		
All-cause mortality						
model 1 (unadjusted)	1	0.85 (0.68, 1.07)	0.84 (0.69, 1.02)	0.76 (0.61, 0.95)	0.92 (0.86, 0.99)	0.02
model 2 (adjusted for age and PIR)	1	0.76 (0.63, 0.92)	0.67 (0.56, 0.79)	0.71 (0.57, 0.87)	0.89 (0.83, 0.96)	0.001
model 3 (multivariate)	1	0.86 (0.69, 1.05)	0.84 (0.69, 1.02)	0.82 (0.63, 1.05)	0.94 (0.87, 1.01)	0.11
Heart disease mortality						
model 1 (unadjusted)	1	0.99 (0.67, 1.45)	0.84 (0.58, 1.23)	0.55 (0.34, 0.89)	0.93 (0.82, 1.06)	0.30
model 2 (adjusted for age and PIR)	1	0.84 (0.57, 1.23)	0.75 (0.48, 1.17)	0.54 (0.32, 0.92)	0.89 (0.78, 1.01)	0.07
model 3 (multivariate)	1	0.72 (0.48, 1.08)	0.88 (0.60, 1.27)	0.84 (0.51, 1.41)	0.96 (0.82, 1.12)	0.60
Cerebrovascular disease mortality						
model 1 (unadjusted)	1	0.62 (0.26, 1.46)	0.99 (0.47, 2.10)	0.75 (0.34, 1.67)	0.96 (0.74, 1.23)	0.72
model 2 (adjusted for age and PIR)	1	0.53 (0.23, 1.21)	0.70 (0.33, 1.50)	0.68 (0.32, 1.47)	0.91 (0.70, 1.19)	0.51
model 3 (multivariate)	1	0.60 (0.24, 1.46)	0.94 (0.44, 2.03)	0.67 (0.32, 1.37)	0.89 (0.69, 1.16)	0.39
Total flavones (mg/day)	<0.18	0.180–0.505	0.505–1.085	≥ 1.085		
Case number (*n*)	3,547	3,493	3,494	3,495		
Person-years	25,298	27,744	27,853	26,870		
All-cause mortality						
Model 1 (unadjusted)	1	0.84 (0.68, 1.02)	0.76 (0.64, 0.92)	0.62 (0.52, 0.74)	0.86 (0.81, 0.91)	<0.001
Model 2 (adjusted for age and PIR)	1	0.75 (0.62, 0.91)	0.72 (0.58, 0.90)	0.64 (0.53, 0.77)	0.87 (0.82, 0.93)	<0.001
Model 3 (multivariate)	1	0.79 (0.61, 1.01)	0.82 (0.62, 1.07)	0.83 (0.66, 1.04)	0.94 (0.88, 1.02)	0.12
Heart disease mortality						
Model 1 (unadjusted)	1	0.99 (0.67, 1.45)	0.84 (0.58, 1.23)	0.55 (0.34, 0.89)	0.83 (0.73, 0.95)	0.01
Model 2 (adjusted for age and PIR)	1	0.84 (0.57, 1.23)	0.75 (0.48, 1.17)	0.54 (0.32, 0.92)	0.83 (0.70, 0.97)	0.02
Model 3 (multivariate)	1	0.85 (0.52, 1.39)	0.86 (0.51, 1.43)	0.63 (0.34, 1.15)	0.89 (0.74, 1.06)	0.19
Cerebrovascular disease mortality						
Model 1 (unadjusted)	1	1.35 (0.63, 2.90)	0.78 (0.39, 1.58)	0.66 (0.35, 1.25)	0.84 (0.70, 0.99)	0.04
Model 2 (adjusted for age and PIR)	1	1.12 (0.53, 2.37)	0.70 (0.36, 1.37)	0.68 (0.34, 1.39)	0.85 (0.68, 1.05)	0.13
Model 3 (multivariate)	1	1.10 (0.46, 2.64)	0.86 (0.46, 1.60)	0.91 (0.44, 1.89)	0.92 (0.75, 1.12)	0.41
Total anthocyanidins (mg/day)	< 0.110	0.110–2.020	2.020–10.775	≥ 10.775		
Case number (*n*)	3,525	3,494	3,504	3,506		
Person-years	25,713	27,449	27,978	26,626		
All-cause mortality						
Model 1 (unadjusted)	1	0.92 (0.75, 1.13)	0.90 (0.74, 1.10)	0.85 (0.68, 1.07)	0.95 (0.89, 1.02)	0.14
Model 2 (adjusted for age and PIR)	1	0.73 (0.61, 0.89)	0.64 (0.52, 0.78)	0.59 (0.48, 0.74)	0.84 (0.78, 0.91)	<0.001
Model 3 (multivariate)	1	0.85 (0.68, 1.06)	0.77 (0.62, 0.95)	0.72 (0.54, 0.96)	0.91 (0.84, 0.99)	0.03
Heart disease mortality						
Model 1 (unadjusted)	1	0.98 (0.62, 1.53)	0.92 (0.59, 1.45)	0.91 (0.61, 1.36)	0.97 (0.86, 1.09)	0.58
Model 2 (adjusted for age and PIR)	1	0.74 (0.49, 1.13)	0.60 (0.39, 0.92)	0.56 (0.36, 0.86)	0.82 (0.72, 0.94)	0.01
Model 3 (multivariate)	1	0.87 (0.55, 1.39)	0.65 (0.40, 1.08)	0.70 (0.41, 1.18)	0.90 (0.78, 1.05)	0.19
Cerebrovascular disease mortality						
Model 1 (unadjusted)	1	1.97 (0.81, 4.78)	1.41 (0.67, 2.94)	0.83 (0.43, 1.63)	0.91 (0.78, 1.07)	0.27
Model 2 (adjusted for age and PIR)	1	1.43 (0.60, 3.38)	0.86 (0.39, 1.88)	0.48 (0.23, 0.98)	0.76 (0.62, 0.93)	0.01
Model 3 (multivariate)	1	1.28 (0.53, 3.09)	0.92 (0.42, 2.01)	0.58 (0.29, 1.13)	0.85 (0.70, 1.03)	0.1
Total flavanones (mg/day)	<0.055	0.055–0.61	0.61–18.95	≥ 18.95		
Case number (*n*)	3,513	3,516	3,493	3,507		
Person-years	24,704	26,457	28,640	27,965		
All-cause mortality						
Model 1 (unadjusted)	1	0.75 (0.60, 0.95)	0.74 (0.56, 0.99)	1.06 (0.85, 1.33)	1.02 (0.94, 1.11)	0.61
Model 2 (adjusted for age and PIR)	1	0.75 (0.57, 0.98)	0.73 (0.55, 0.95)	0.83 (0.64, 1.07)	0.95 (0.87, 1.03)	0.20
Model 3 (multivariate)	1	0.88 (0.65, 1.18)	0.91 (0.66, 1.24)	1.06 (0.81, 1.41)	1.03 (0.94, 1.12)	0.57
Heart disease mortality						
Model 1 (unadjusted)	1	0.63 (0.41, 0.96)	0.81 (0.48, 1.37)	0.84 (0.58, 1.20)	0.97 (0.84, 1.11)	0.63
Model 2 (adjusted for age and PIR)	1	0.61 (0.38, 0.97)	0.73 (0.45, 1.18)	0.58 (0.41, 0.84)	0.86 (0.76, 0.98)	0.03
Model 3 (multivariate)	1	0.77 (0.45, 1.32)	0.90 (0.52, 1.58)	0.72 (0.49, 1.07)	0.94 (0.83, 1.07)	0.34
Cerebrovascular disease mortality						
Model 1 (unadjusted)	1	1.18 (0.48, 2.89)	1.08 (0.44, 2.68)	3.09 (1.02, 9.35)	1.51 (1.00, 2.26)	0.05
Model 2 (adjusted for age and PIR)	1	1.14 (0.47, 2.73)	0.98 (0.39, 2.46)	2.08 (0.73, 5.89)	1.30 (0.91, 1.86)	0.15
Model 3 (multivariate)	1	1.50 (0.51, 4.38)	1.23 (0.43, 3.53)	2.69 (0.77, 9.40)	1.35 (0.95, 1.91)	0.09
Total flavonols (mg/day)	<6.815	6.815–12.555	12.555–22.105	≥22.105		
Case number (*n*)	3,511	3,504	3,507	3,507		
Person-years	25,931	26,744	27,285	27,806		
All-cause mortality						
Model 1 (unadjusted)	1	0.74 (0.65, 0.84)	0.67 (0.54, 0.83)	0.54 (0.45, 0.65)	0.82 (0.77, 0.87)	<0.001
Model 2 (adjusted for age and PIR)	1	0.75 (0.68, 0.83)	0.72 (0.60, 0.86)	0.65 (0.52, 0.80)	0.87 (0.81, 0.93)	<0.001
Model 3 (multivariate)	1	0.81 (0.71, 0.93)	0.74 (0.61, 0.90)	0.66 (0.53, 0.84)	0.87 (0.81, 0.94)	<0.001
Heart disease mortality						
Model 1 (unadjusted)	1	0.94 (0.63, 1.41)	0.74 (0.49, 1.10)	0.56 (0.33, 0.93)	0.82 (0.71, 0.94)	0.01
Model 2 (adjusted for age and PIR)	1	0.94 (0.63, 1.39)	0.78 (0.51, 1.19)	0.68 (0.40, 1.13)	0.87 (0.75, 1.01)	0.07
Model 3 (multivariate)	1	1.00 (0.64, 1.57)	0.83 (0.51, 1.34)	0.74 (0.42, 1.31)	0.90 (0.76, 1.07)	0.25
Cerebrovascular disease mortality						
Model 1 (unadjusted)	1	0.98 (0.36, 2.65)	0.75 (0.32, 1.73)	0.51 (0.24, 1.07)	0.80 (0.65, 0.98)	0.03
Model 2 (adjusted for age and PIR)	1	0.99 (0.35, 2.78)	0.81 (0.37, 1.77)	0.68 (0.32, 1.45)	0.88 (0.72, 1.07)	0.19
Model 3 (multivariate)	1	0.96 (0.32, 2.85)	0.56 (0.23, 1.41)	0.57 (0.26, 1.24)	0.81 (0.64, 1.01)	0.06
Total flavan-3-ols (mg/day)	<4.915	4.915–15.425	15.425–154.295	≥154.295		
Case number (*n*)	3,511	3,504	3,507	3,507		
Person-years	26,560	27,212	25,928	28,065		
All-cause mortality						
Model 1 (unadjusted)	1	1.01 (0.85, 1.21)	0.88 (0.70, 1.10)	0.85 (0.70, 1.03)	0.94 (0.88, 1.00)	0.05
Model 2 (adjusted for age and PIR)	1	0.87 (0.75, 1.00)	0.71 (0.59, 0.86)	0.77 (0.63, 0.94)	0.91 (0.85, 0.97)	0.01
Model 3 (multivariate)	1	0.98 (0.82, 1.17)	0.85 (0.69, 1.05)	0.87 (0.69, 1.08)	0.95 (0.88, 1.02)	0.13
Heart disease mortality						
Model 1 (unadjusted)	1	1.32 (0.89, 1.97)	1.08 (0.73, 1.60)	1.00 (0.68, 1.48)	0.97 (0.87, 1.09)	0.65
Model 2 (adjusted for age and PIR)	1	1.08 (0.76, 1.52)	0.81 (0.55, 1.20)	0.87 (0.61, 1.25)	0.93 (0.83, 1.04)	0.21
Model 3 (multivariate)	1	1.28 (0.89, 1.84)	1.01 (0.67, 1.50)	1.10 (0.73, 1.65)	1.00 (0.88, 1.13)	0.98
Cerebrovascular disease mortality						
Model 1 (unadjusted)	1	0.95 (0.41, 2.20)	0.80 (0.33, 1.95)	0.72 (0.36, 1.46)	0.89 (0.72, 1.10)	0.29
Model 2 (adjusted for age and PIR)	1	0.76 (0.34, 1.70)	0.59 (0.22, 1.57)	0.63 (0.32, 1.26)	0.85 (0.67, 1.08)	0.18
Model 3 (multivariate)	1	0.83 (0.36, 1.92)	0.70 (0.29, 1.71)	0.52 (0.25, 1.05)	0.81 (0.65, 1.01)	0.06

Hazard ratios (95% CI) for all-cause, heart disease, and cerebrovascular disease mortality were analyzed using Cox proportional hazard models. Model 1 was unadjusted; Model 2 was adjusted for age and PIR; and Model 3 was adjusted for age, PIR, sex, education status, race, daily energy intake, BMI, smoking status, alcohol intake, total time of PA, and hyperlipidemia.

PIR, poverty income ratio; BMI, body mass index; PA, physical activity.

As the total intake of isoflavones was zero in 37.92% (5,320) of participants, the cohort was divided into two groups based on the median intake of isoflavones. We found that isoflavone intake was inversely correlated with all-cause mortality (0.81 [0.70, 0.94], *p* = 0.01, Table 4). The intake of isoflavones was tended to be associated with reduced heart disease mortality (0.75 [0.56,1.01], *p* = 0.06).

**Table 4:** Hazard ratios of mortality by isoflavone intake groups.

Total isoflavones (mg/day)	< 0.010	≥ 0.010	
Case number (*n*)	7,187	6,842	
Person-years	55,194	52,571	
		HR (95%CI)	*p* value
All-cause mortality			
Model 1 (unadjusted)	1	0.66 (0.58, 0.76)	<0.001
Model 2 (adjusted for age and PIR)	1	0.74 (0.64, 0.86)	<0.001
Model 3 (multivariate)	1	0.81 (0.70, 0.94)	0.01
Heart disease mortality			
Model 1 (unadjusted)	1	0.62 (0.47, 0.81)	<0.001
Model 2 (adjusted for age and PIR)	1	0.70 (0.53, 0.91)	0.01
Model 3 (multivariate)	1	0.75 (0.56, 1.01)	0.06
Cerebrovascular disease mortality			
Model 1 (unadjusted)	1	1.09 (0.69, 1.71)	0.71
Model 2 (adjusted for age and PIR)	1	1.26 (0.78, 2.03)	0.34
Model 3 (multivariate)	1	1.30 (0.76, 2.20)	0.34

Hazard ratios (95% CI) for all-cause, heart disease, and cerebrovascular disease mortality were analyzed using Cox proportional hazard models. Model 1 was unadjusted; Model 2 was adjusted for age and PIR; and Model 3 was adjusted for age, PIR, sex, education status, race, BMI, daily energy intake, smoking status, alcohol intake, total time of PA, and hyperlipidemia.

PIR, poverty income ratio; BMI, body mass index; PA, physical activity.

**Figure 2:**
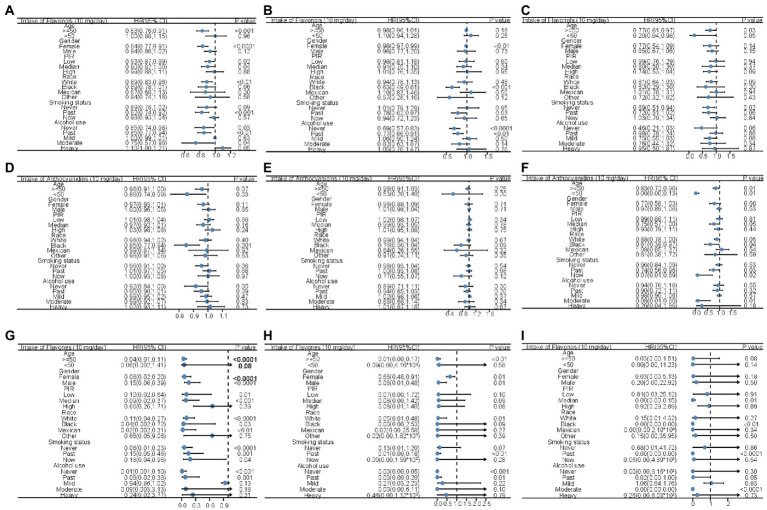
Forest plots displaying the association between flavonol intake and all-cause mortality **(A)**, mortality of heart diseases **(B)**, and mortality of cerebrovascular diseases **(C)**; the association between anthocyanidin intake and all-cause mortality **(D)**, mortality of heart diseases **(E)**, and mortality of cerebrovascular diseases **(F)**; and the association between flavones intake and all-cause mortality **(G)**, mortality of heart diseases **(H)**, and mortality of cerebrovascular diseases **(I)** in unadjusted Cox analysis stratified by age, gender, PIR, race, smoking status, and alcohol usage with the increment unit of 10 mg/day. HR: Hazard ratios; PIR: poverty income ratio.

To explore how the stratification factors, including age, sex, PIR, race, smoking status and drinking status, were associated with mortality, stratified Cox models were conducted with the increment unit of 10 mg/day (Figure 2; Supplementary Figure 2). The intake of total flavonols was associated with reduced all-cause mortality in most groups, especially in the groups aged 50 and above, in females, in people with low or medium PIR, in whites, former smokers, non-drinkers, former drinkers, and moderate alcohol users (Figure 2A). Moreover, similar results were observed that the intake of total flavonols was associated with reduced heart disease mortality in those that were female, black, former smokers, and former drinkers, as well as in those who never drank (Figure 2B). The total intake of flavonols was negatively associated with mortality from cerebrovascular diseases in all age groups and in people who never smoked (Figure 2C).

The inverse association between mortality and total anthocyanidin intake was significant in that those were younger than 50 years, black, and in people who never drink (Figure 2D). There was a trend that the intake of total anthocyanidins was negatively associated with reduced mortality caused by heart disease in black people (Figure 2E). The total anthocyanidin intake was significantly negatively associated with cerebrovascular mortality in all age groups, in people with medium PIR, in white and black people, in former and current smokers, and in moderate alcohol users (Figure 2F).

The increased intake of total flavones was associated with a reduction in the possibility of all-cause death in much of the population, but not the people younger than 50 years, high PIR, other races, and current alcohol users (Figure 2G). Similar results were observed in that the intake of total flavones was inversely associated with a reduced risk of death caused by heart disease in the group aged >50 years, in both sexes, in white people, in former smokers, and in people who never drank or were used to drinking (Figure 2H). In addition, mortality related to cerebrovascular disease was inversely associated with the intake of total flavones in people with medium PIR, in black people, in former smokers, and in former and moderate alcohol users (Figure 2I).

The stratified association between mortality and total flavonoid, flavanone, isoflavone, and flavan-3-ol intake with the increment unit of 10 mg/day is shown in Supplementary Figure S2. The intake of flavanones was positively associated with all-cause mortality in females, people with high PIR, whites, non-smokers, and former drinkers (Supplementary Figure S2A). The inverse association between total flavanone intake and heart disease mortality was seen in people younger than 50 and current smokers (Supplementary Figure S2E). However, the intake of flavanones was positively associated with the cerebrovascular disease mortality in people age 50 and above, both genders, people with high PIR, whites, non-smokers, former smokers, and mild alcohol users (Supplementary Figure S2I). Total isoflavone intake and all-cause mortality were negatively associated in former smokers and people with high PIR (Supplementary Figure S2B). The intake of isoflavone did not show inverse association with heart disease mortality in any subgroups (Supplementary Figure S2F). The inverse association between total isoflavone intake and cerebrovascular mortality was significant in males, people with medium PIR, current smokers, and mild alcohol users, and former alcohol users (Supplementary Figure S2J). In the female participants and former drinkers, the intake of flavan-3-ols was inversely associated with all-cause mortality (Supplementary Figure S2C). The intake of flavan-3-ols was inversely associated with heart disease mortality in females, blacks, people never drank, and former drinkers, while the intake of flavan-3-ols was positively associated with heart disease mortality in mild alcohol users (Supplementary Figure S2G). The cerebrovascular disease mortality was inversely associated with the intake of flavan-3-ols in males and non-alcohol users (Supplementary Figure S2K). The reduction in all-cause mortality was only associated with total flavonoid intake in people aged 50 and above, female, and people who used to drinking alcohol (Supplementary Figure S2D). In the female participants, blacks, non-alcohol users, and former drinkers, the intake of total flavonoids was inversely associated with heart disease mortality, while the intake of flavonoids was positively associated with heart disease mortality in mild alcohol users (Supplementary Figure S2H). The inverse association between intake of total flavonoid and cerebrovascular disease mortality was only observed in the male participants (Supplementary Figure S2L). Notably, some of the effects of an increment mentioned above were rather small, and those results should be interpreted with caution.

### 3.3. Correlation among flavonoid intakes and correlation between isoflavone intake and urinary phytoestrogens

**Figure 3:**
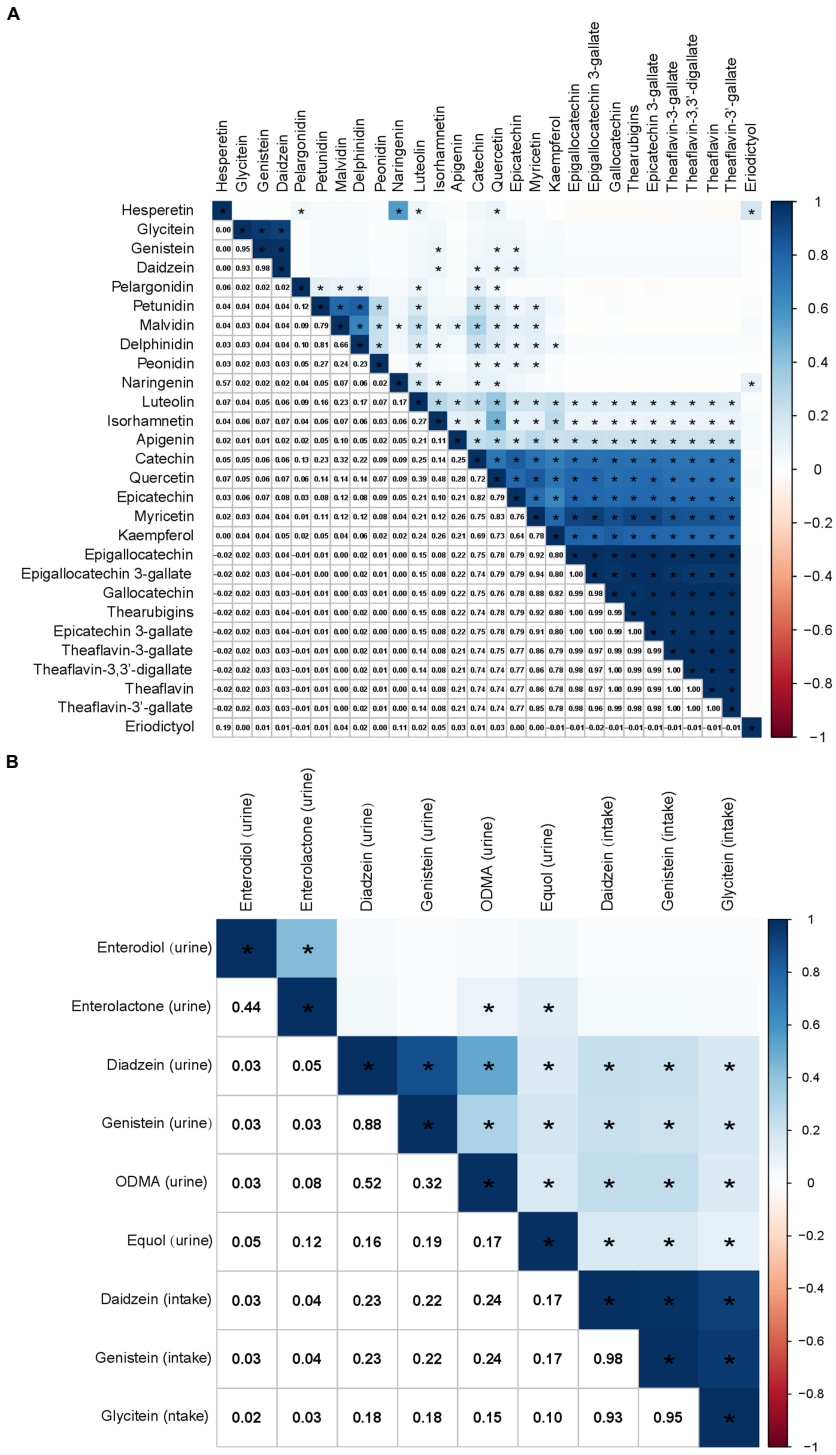
Correlations among flavonoid intakes **(A)** and the correlation between isoflavone intake and urinary phytoestrogens **(B)**, calculated by Pearson’s method. The numbers in the lower left half of the correlation graph represent the Pearson’s correlation coefficients. The color bars represent the Pearson’s correlation coefficients, dark blue as positive correlation and red as negative correlation. * *p* < 0.0001.

The correlations among flavonoid intake levels are shown in Figure 3A. There was a strong correlation between genistein and glycitein (*r* = 0.95), daidzein and glycitein (*r* = 0.93), and daidzein and genistein (*r* = 0.98). There was also a high or moderate correlation between flavonols, including quercetin and myricetin (*r* = 0.83), quercetin and kaempferol (*r* = 0.73), and myricetin and kaempferol (*r* = 0.78). Flavan-3-ols intake was highly or moderately correlated with each other (Figure 3A). The intake of flavonols was highly or moderately correlated with the intake of flavan-3-ols (Figure 3A).

At the same time, there was a weak association between the consumption of daidzein and genistein and their metabolites in urine (Figure 3B). However, there was no correlation between glycitein intake and the urinary phytoestrogen levels (Figure 3B). The correlations between other flavonoids and urinary phytoestrogens are shown in Supplementary Figure S3.

### 3.4. Construction and validation of risk score

To make it easier to use flavonoid intake to assess mortality risk and improve the lifestyle of the population, a univariate Cox hazards regression analysis was employed to screen survival-related flavonoids. The results showed that the consumed amounts of specific flavonoids, including total flavones, total flavanones, total flavonols, eriodictyol, luteolin, isorhamnetin, quercetin, naringenin, kaempferol, and apigenin, were significantly correlated with life expectancy. The strength of the correlation for each flavonoid is summarized in Supplementary Table S3. To avoid multicollinearity, the risk score model was constructed using intake values of single flavonoids, including eriodictyol, luteolin, isorhamnetin, quercetin, naringenin, kaempferol, and apigenin in both the training and testing datasets. The cohort was divided into low- and high-risk groups, based on the median risk score. The relevant baseline variables were further analyzed based on the risk groups (Table 5). The participants in the high-risk group were younger (45.84 ± 0.37, *p* < 0.0001), female (55.75%, *p* < 0.0001), had lower education levels, lower PIR (2.70 ± 0.05, *p* < 0.0001), higher BMI (29.59 ± 0.16), lower HEI score (49.13 ± 0.33, *p* = 0.0001), higher DII (2.20 ± 0.04, *p* < 0.0001), current smokers (22.63%, *p* < 0.0001), and most had cardiovascular diseases, asthma/COPD, and stroke (*p* = 0.03, *p* = 0.02, *p* = 0.001). However, participants in the high-risk group were less frequently current alcohol users (*p* < 0.0001).

**Table 5:** Relevant baseline variables according to the risk group.

	High risk	Low risk	*p* value
Baseline sociodemographic, lifestyle, and health-related variables			
Age, years	45.84 ± 0.37	48.05 ± 0.42	<0.0001
Sex, %			<0.0001
Female	55.75 (54.18, 57.32)	50.10 (48.53, 51.67)	
Male	44.25 (42.68, 45.82)	49.90 (48.33, 51.47)	
Race, %			<0.0001
Black	14.78 (12.17, 17.38)	8.64 (7.26, 10.01)	
Mexican	9.28 (7.01, 11.55)	8.10 (6.11, 10.08)	
Other	12.14 (10.23, 14.05)	13.79 (11.43, 16.16)	
White	63.81 (59.56, 68.06)	69.47 (65.63, 73.31)	
Education, %			<0.0001
1	6.75 (5.77, 7.73)	3.52 (2.90, 4.13)	
2	12.92 (11.53, 14.31)	8.75 (7.80, 9.69)	
3	28.85 (26.66, 31.04)	21.60 (20.00, 23.21)	
4	30.85 (29.27, 32.42)	29.63 (27.63, 31.64)	
5	20.63 (18.39, 22.87)	36.50 (33.44, 39.56)	
Marital status, %			<0.0001
1	51.02 (48.81, 53.23)	58.64 (56.03, 61.25)	
2	10.34 (9.32, 11.36)	9.39 (8.46, 10.31)	
3	2.59 (2.05, 3.12)	2.38 (1.93, 2.82)	
4	21.42 (19.58, 23.26)	17.03 (15.30, 18.76)	
5	6.77 (5.90, 7.64)	4.94 (4.38, 5.50)	
6	7.86 (7.02, 8.70)	7.63 (6.28, 8.98)	
PIR	2.70 ± 0.05	3.30 ± 0.05	<0.0001
BMI (kg/m^2^)	29.59 ± 0.16	28.75 ± 0.16	<0.0001
Total score of HEI	49.13 ± 0.33	56.77 ± 0.43	0.0001
DII	2.20 ± 0.04	0.86 ± 0.05	<0.0001
Total time of PA (mins/week)	1,304.14 ± 39.84	1,258.18 ± 46.92	0.42
Total MET of PA (/week)	5,216.56 ± 159.38	5,032.71 ± 187.67	0.42
Smoking status, %			<0.0001
Former	22.74 (21.20, 24.27)	25.84 (23.91, 27.77)	
Never	54.63 (52.21, 57.06)	58.24 (55.87, 60.61)	
Now	22.63 (20.64, 24.61)	15.92 (14.52, 17.33)	
Alcohol usage, %			<0.0001
Former	13.21 (11.56, 14.87)	9.44 (8.06, 10.83)	
Heavy	22.60 (20.74, 24.46)	21.23 (19.61, 22.85)	
Mild	34.60 (31.88, 37.33)	41.27 (39.33, 43.20)	
Moderate	16.78 (15.62, 17.93)	19.10 (17.48, 20.73)	
Never	12.81 (11.53,14.09)	8.95 (7.83, 10.08)	
Disease diagnosis at interview			
Hyperlipidemia, %			0.65
No	30.98 (28.79, 33.17)	31.55 (29.56, 33.55)	
Yes	69.02 (66.83, 71.21)	68.45 (66.45, 70.44)	
Cardiovascular disease, %			0.03
No	90.47 (89.30, 91.65)	91.82 (90.77, 92.86)	
Yes	9.53 (8.35, 10.70)	8.18 (7.14, 9.23)	
Respiratory system disease, %			0.02
ACO	2.50 (2.02, 2.99)	1.87 (1.30, 2.43)	
Asthma	12.99 (11.54, 14.43)	10.69 (9.55, 11.83)	
COPD	3.09 (2.55, 3.62)	3.02 (2.30, 3.75)	
No	81.42 (79.82, 83.02)	84.42 (82.84, 86.00)	
Stroke, %			0.001
No	95.97 (95.34, 96.60)	97.31 (96.80, 97.82)	
Yes	4.03 (3.40, 4.66)	2.69 (2.18, 3.20)	
Cancer, %			0.26
No	90.67 (89.73, 91.60)	89.74 (88.55, 90.93)	
Yes	9.33(8.40,10.27)	10.26 (9.07, 11.45)	
Hypertension, %			0.19
No	62.46 (60.37, 64.55)	63.97 (61.84, 66.09)	
Yes	37.54 (35.45, 39.63)	36.03 (33.91, 38.16)	
DM, %			0.3
No	78.39 (76.61, 80.17)	79.47 (77.82, 81.11)	
Yes	21.61 (19.83, 23.39)	20.53 (18.89, 22.18)	

Continuous variables are presented as mean ± standard deviation and categorical variables are presented as percentage (95% confidence intervals, 95% CIs). PIR, poverty income ratio; BMI, body mass index; HEI, Healthy Eating Index 2015 version; DII, dietary inflammatory index; PA, physical activity; MET, metabolic equivalent; COPD, chronic obstructive pulmonary disease; DM, type 2 diabetes mellitus. In education, less than 9th grade was coded as 1; 9–11th grade (includes 12th grade with no diploma) as 2, high school graduation or equivalent as 3, some college or associates degree as 4, and college graduate or above as 5. For marital status, married was coded as 1, divorced as 2, separated as 3, never married as 4, widowed as 5, and living with partner as 6.

**Figure 4:**
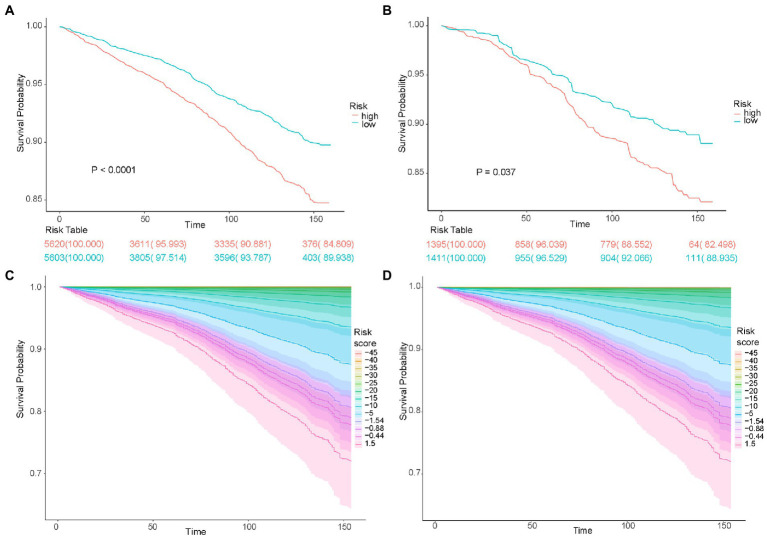
The survival time of patients according to the risk score. Kaplan–Meier analyses of survival time based on risk group in the training cohort **(A)** and testing cohort **(B)**. Kaplan–Meier analyses of survival time based on different risk score levels in the training cohort **(C)** and testing cohort **(D)**.

The utility of the risk score was validated using the Kaplan–Meier survival curve. The survival probabilities were significantly lower in both the training dataset (*p* < 0.0001) and the testing dataset (*p* = 0.037) in the high-risk group than in the low-risk group (Figures 4A,B). The risk score was inversely associated with survival probabilities in both the training and testing datasets after adjusting for PIR and age (Figures 4C,D).

**Figure 5:**
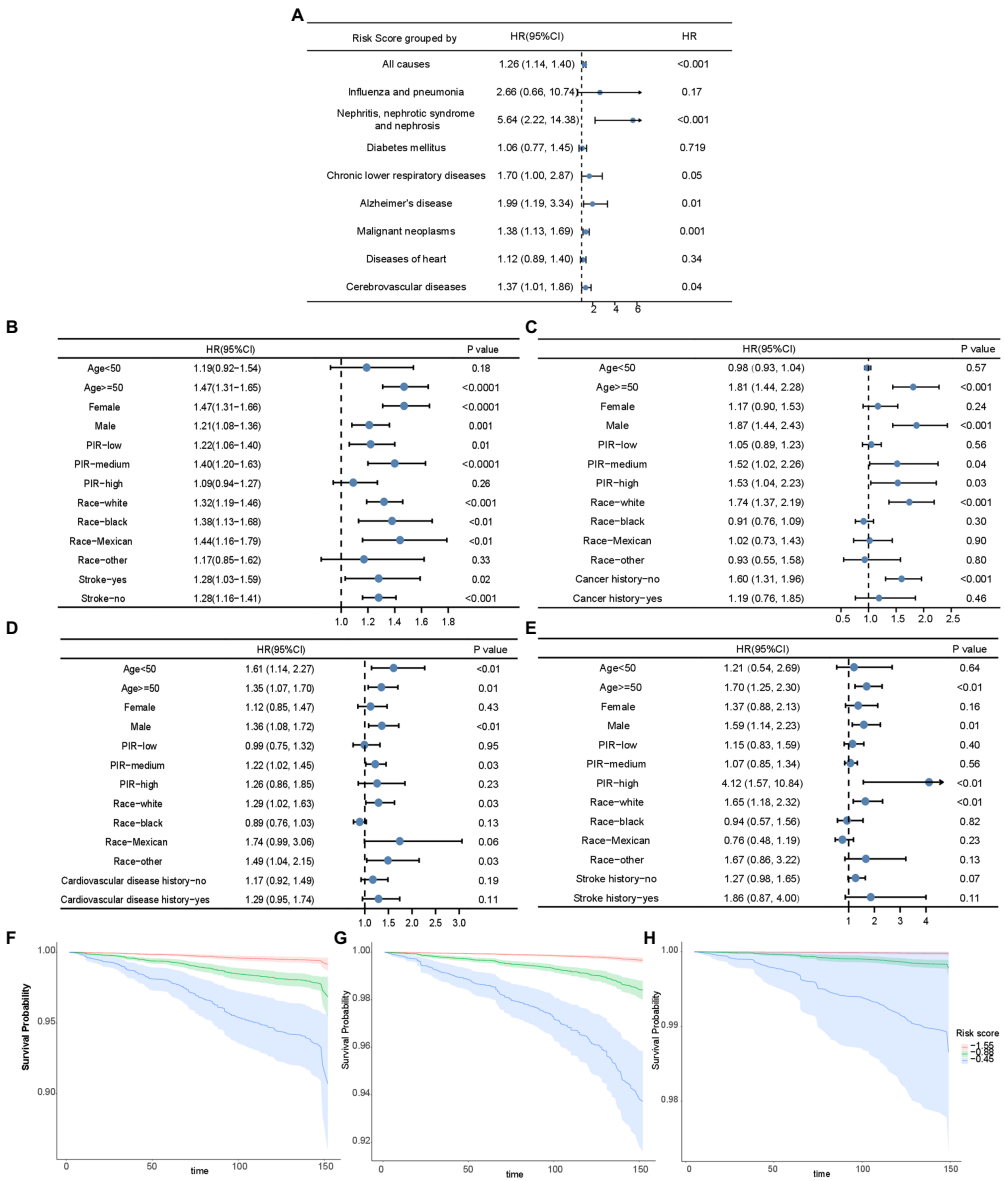
The association of risk score and mortality. **(A)** The forest plot displaying the association between risk score and all-cause mortality and disease-specific mortality in Cox analysis adjusted for age and PIR. The forest plots demonstrating the association between risk score and all-cause mortality **(B)**, cancer-related mortality **(C)**, mortality of heart diseases **(D)**, and mortality of cerebrovascular diseases **(E)** in unadjusted Cox analysis stratified by age, sex, PIR, race, and disease history. Kaplan–Meier curves based on risk score quartiles to demonstrate the death possibility caused by cancer **(F)**, heart diseases **(G)**, and cerebrovascular diseases **(H)**, after adjustment for PIR and age. HR: Hazard ratios; PIR: poverty income ratio.

Multivariate adjusted Cox proportional hazard analyses revealed a positive correlation between risk score and the total mortality 1.26 [1.14–1.40]; *p* < 0.001), death caused by nephritis, nephrotic syndrome, and nephrosis (5.64 [2.22–14.38]; *p* < 0.001), mortality related to chronic lower respiratory diseases (1.70 [1.00–2.87]; *p* = 0.05), mortality related to Alzheimer’s disease (1.99 [1.19–3.34]; *p* = 0.01), mortality of malignant neoplasms (1.38 [1.13–1.69]; *p* = 0.001), death caused by heart diseases (1. 12 [0.89–1.40]; *p* = 0.34), and mortality caused by cerebrovascular disease (1.37 [1.01–1.86]; *p* = 0.04) (Figure 5A). Subsequent Cox models were stratified according to age, sex, PIR, race, and disease history (Figures 5B–E). The performance of the risk score for predicting all-cause mortality was relatively robust in most stratified groups, except in individuals younger than 50 years, with high PIR, and in other minority races (Figure 5B). In addition, there was a positive association between the risk score and cancer-related death in participants older than 50 years, males, participants with medium and high PIR, and those without a history of cancer (Figure 5C). There was a positive association between the risk score and heart disease mortality in all age groups, males, groups with medium PIR, whites, and other races (Figure 5D). Moreover, the risk score was positively associated with the cerebrovascular disease mortality in the participants ≥50 years, males, in the group with high PIR, in the white people (Figure 5E). As shown in Figures 5G–I, the risk score was positively associated with mortality caused by cancer, heart diseases, and cerebrovascular diseases after adjustment for PIR and age. Therefore, the risk score can be used to estimate survival probabilities in the population.

### 3.5. Establishment and validation of nomogram with risk score

**Figure 6:**
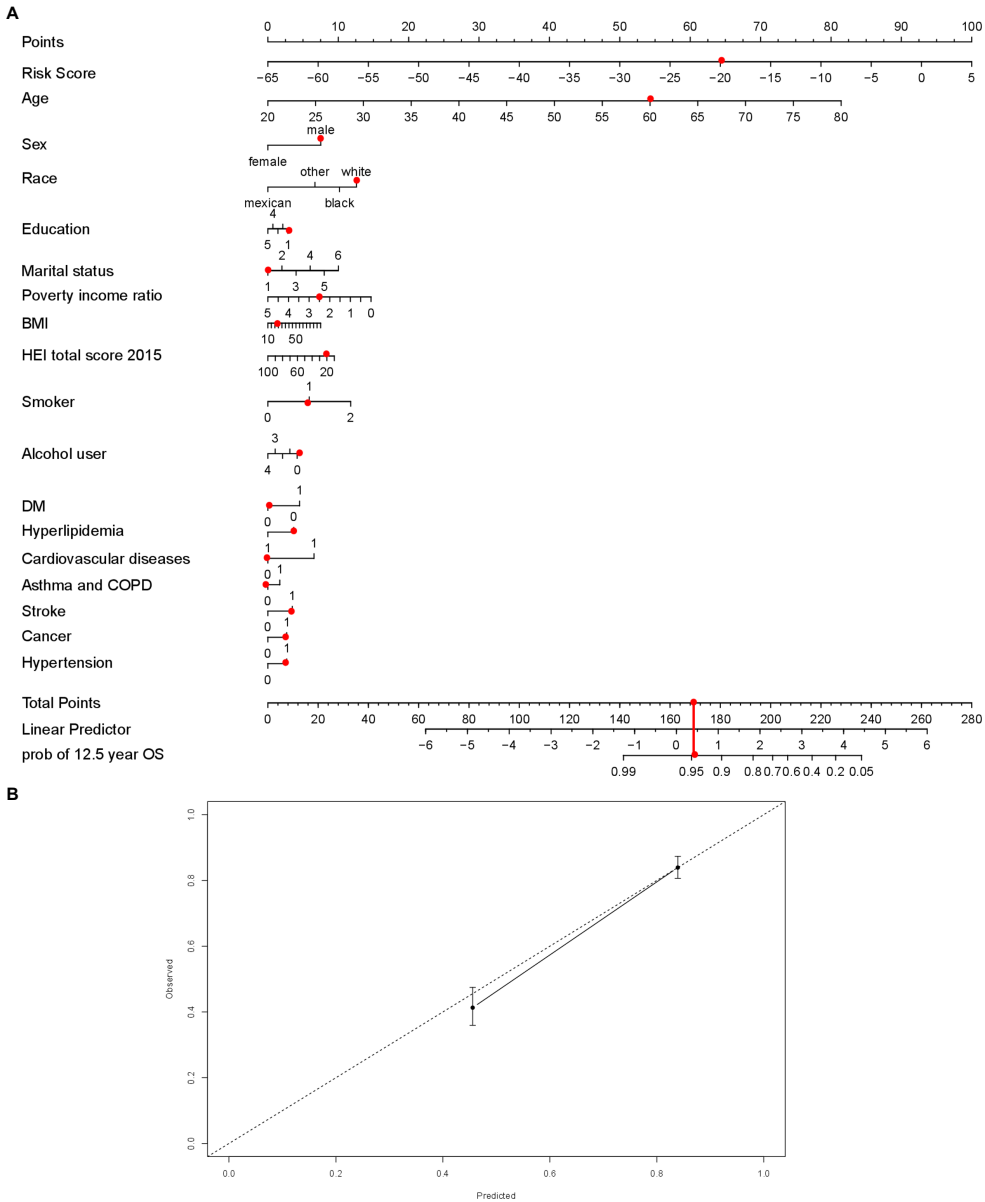
The nomogram established with risk score **(A)**, red dots demonstrated an example with points for each variables, total points and possibility of survival at 12.5 years. Calibration curve to assess the consistency of predicted survival possibility at 12.5 years by the nomogram with risk score. **(B)** BMI = body mass index; HEI = Healthy Eating Index, 2015 version; Smoking indicated as 0 (non-smokers), 1 (former smokers), and 2 (current smokers); Alcohol use indicated as 0 (non-drinkers), 1 (former drinkers), 2 (mild drinkers), 3 (moderate drinkers), and 4 (heavy drinkers); COPD = chronic obstructive pulmonary disease; DM = type 2 diabetes mellitus. In the disease prevalence, 0 indicates no and 1 indicates yes. In the education, less than 9th grade was coded as 1; 9–11th grade (includes 12th grade with no diploma) as 2; high school graduation or equivalent as 3; some college or associates degree as 4; college graduate or above as 5. For the marital status, married was coded as 1; divorced as 2; separated as 3; never married as 4; widowed as 5; living with partner as 6.

We then built an easy-to-use and clinically adaptable risk nomogram for predicting the survival probability in 12.5 years (Figure 6A). For instance, a white (12.60 points) married (0 points) male (7.52 points), 60 years old (54.29 points), an ex-smoker (5.88 points) as well as non-drinker (4.17 points), with risk score −20 (64.29 points), <9-year education (2.85 points), 2.5 of PIR (7.33 points), 20 of BMI (1 point), 20 of HEI (1 point), no history of DM (0 points), hyperlipidemia (3.68 points), cardiovascular disease (0 points), and chronic lower respiratory diseases (0 points), and history of stroke (3.47 points), cancer (2.68 points) and hypertension (2.68 points), who was with a predicted 12.5-year survival possibility was about 95% (total points 173.44, Figure 6A, red dots). A higher total point was associated with a lower 12.5-year survival rate. The predictions made by the nomogram model were close to the observed outcomes of 12.5-year survival (Figure 6B).

## 4. Discussion

A healthy diet reduces the risk of developing non-communicable diseases (NCDs), such as heart disease, cerebrovascular disease, and cancer. Approximately 8.4% of NCD mortality is associated with low intake of fruits or vegetables (22). In addition, the diet for disease prevention is modifiable, and people can adopt personalized dietary patterns that target unhealthy lifestyles and disease history. Increasing evidence has shown that the intake of flavonoids and flavonoid-rich foods is inversely associated with the risk of all-cause mortality (22). Vegetables such as broccoli, garlic, and onions are rich in flavonols, including isorhamnetin, quercetin, kaempferol, and myricetin (23). Flavanones such as eriodictyol, hesperetin, and naringenin are readily present in large amounts in citrus fruits (23). Celery, parsley, artichokes, chicory, tea, and herb leaves are rich in flavones, including luteolin and apigenin (23). Anthocyanidins, including cyanidin, delphinidin, malvidin, pelargonidin, peonidin, and petunidin, are present in blueberries, raspberries, and red wine (23). Additionally, flavan-3-ols are present in green and black teas, red wine, chocolate, and apricots (24).

We found lower all-cause mortality was associated with anthocyanidin intake, which is in agreement with previous findings (22, 23, 25). In this stratified study, anthocyanidin intake was inversely associated with all-cause and cerebrovascular mortality in black individuals. Anthocyanidin intake was significantly associated with decreased cerebrovascular mortality in both former and current smokers. Smoking is a major risk factor for strokes. In addition, anthocyanidin intake was inversely associated with cerebrovascular mortality in moderate alcoholics and participants aged 50 years and above. Research has demonstrated an inverse association between anthocyanidin intake and stroke mortality, as well as stroke incidence (26). Moreover, there is plenty of evidence of an inverse association between cardiovascular mortality and anthocyanidin intake (10, 23, 26, 27). *In vitro* studies have shown that the main action site of anthocyanins is the vascular endothelium, which improves vascular tone (28). Anthocyanidins can improve carbohydrate metabolism and insulin sensitivity, and lower total cholesterol and low-density lipoprotein levels (29).

The inverse association between flavonol intake and all-cause mortality was consistent with a prospective study of middle-aged women (30). It is well documented that flavonols play a protective role in atherosclerosis and hypertension by inhibiting oxidative stress, endothelial dysfunction, and inflammation (31). In analyses stratified by sex, we revealed a significant inverse association between flavonol intake and all-cause mortality and mortality due to heart disease in women, but not in men. These results are in line with those of previous studies (32, 33) reporting the protective effects of antioxidants, particularly in women. Moreover, we observed an inverse association between flavonol intake and all-cause mortality and heart disease mortality in former smokers, former alcohol users, and non-alcohol users. Flavonol intake was inversely associated with cerebrovascular disease in non-smokers (*p* = 0.02). These results suggest that the dietary intake of flavonols may not offset the harmful effects of unhealthy life. Interestingly, quercetin was inversely associated with heart disease mortality after adjusting for age and PIR (0.70 [0.01–0.97]; *p* = 0.03). Quercetin can inhibit the expression matrix metalloproteinases to reduce plaque rupture and subsequent myocardial ischemia (34). Moreover, flavonols can play an important role in reducing the reactive oxygen species overproduced in ischemia/reperfusion and protecting neurons from apoptosis and necrosis in stroke (24, 35).

Although it has been reported that soybean consumption in Western populations is about one-tenth of that in Asians (10), we observed an inverse association between the total intake of isoflavones and total mortality, as well as heart disease mortality (of marginal significance) in Western populations. This result has rarely been described in Western populations (10, 23, 36) but in Asians (37). Interestingly, we found that the weak associations between the intake of isoflavone and phytoestrogens in urine are in agreement with findings that the diverging capability of metabolizing the soy isoflavone daidzein to the more bioactive metabolite equol or ODMA among individuals may be determined by genetics, gut microbiome composition, and dietary pattern (38). Equol- and ODMA-producing pre-hypertensive postmenopausal Chinese women may receive more benefits from isoflavone intake in reducing the risk of cardiovascular diseases (39, 40).

Moreover, the inverse association between flavone intake and all-cause mortality in our study (Figure 1A) agrees with previous findings (41, 42). The significant inverse association between intake of flavones and heart disease mortality were noted in those aged 50 and above, in females, and in former alcoholics. In particular, former smokers may have the greatest benefits of flavones against heart diseases, which might be supported by the evidence that flavones may have a cardioprotective effect in smokers (43). Therefore, the intake of flavones is an important intervention for the elderly and smokers to reduce premature death and heart disease-related mortality.

Another interesting finding was that the intake of flavan-3-ol was tended to be inversely associated with reduced cerebrovascular mortality, which may be supported by a prospective study that men in the highest quartile of flavan-3-ol intake had a decreased incidence of ischemic stroke (0.59, [0.30–1.14]) compared to the lowest quartile (11). A meta-analysis may also support our observation that the intake of flavan-3-ols was associated with a decreased risk of cardiovascular diseases (32). In addition, flavan-3-ols were associated with a lower risk of fatal cardiovascular diseases (all *p*-trend = 0.02) (10). However, it was also reported that there was no association between catechin intake and stroke incidence or mortality (44). Mechanistic studies in humans suggest a protective role for flavan-3-ols in blood pressure regulation and reduction of low-density lipoprotein oxidation by quenching free radicals, chelating metals, or recycling other antioxidants (45, 46, 47).

Contrary to the findings of a previous study (23), we found a positive association between total flavanone intake and all-cause mortality in the unadjusted Cox analysis, as well as cerebrovascular mortality. Ivey et al. (30) also observed that high-flavanone consumers tend to have higher mortality rates. It may be due to consumption of sugar-rich grapefruit juices, one source of flavanones, influencing the effects of flavanones (30). Additionally, grapefruit components interact with drugs independently of their flavonoid content (48). Furthermore, our results are partially in agreement with those obtained in Cassidy’s study for men, in which total flavanone intake tended to be positively associated with hemorrhagic stroke incidence (1.75, [1.09, 2.82], *p* = 0.09). Previous findings on the association between flavanone intake and the risk of ischemic stroke were inconsistent. It has been reported that total flavanone intake decreases the risk of ischemic stroke incidence in female (49). Goetz et al. (50) reported that the highest quintile of intake of flavanones was inversely associated with the incidence of acute ischemic stroke as compared to the lowest quintile. However, Mursu et al. did not find an inverse association between flavanone intake and ischemic stroke incidence (0.89, 0.49–1.63) (11). Few studies have investigated the association between flavanone intake and mortality in cerebrovascular disease. A study on postmenopausal women without a history of cardiovascular disease did not reveal an inverse association between the risk of death from stroke and flavanone intake after adjustment for age and energy (0.88, [0.66, 1.17]) and in the multivariable Cox analysis (0.94, [0.69, 1.27]) (23). In our analysis, cerebrovascular mortality (ICD I60–I69) included not only death caused by ischemic stroke, but also hemorrhagic stroke and other cerebral vascular diseases, such as Moyamoya disease (ICD I67.5). A detailed analysis is warranted to determine whether flavanones have different effects on hemorrhagic and ischemic stroke. Another limitation of our analysis of cerebrovascular mortality was that only 87 deaths occurred. Further longitudinal follow-up is required to determine whether total flavanone intake is associated with death caused by cerebrovascular events.

For the individual flavonoid compounds, we found that the intake of apigenin, luteolin, isorhamnetin, peonidin, and eriodictyol was associated with lower all-cause mortality after adjusting for PIR and age. Notably, eriodictyol intake was associated with decreased mortality due to cerebrovascular diseases. Peonidin intake was associated with lower mortality due to heart disease.

Due to the complex contribution of flavonoids to mortality, we constructed a risk score to evaluate the risk of death. The risk score consisted of eriodictyol, luteolin, isorhamnetin, quercetin, naringenin, kaempferol, and apigenin. It was found to be robust for evaluating all-cause mortality in most populations. Application of the risk score revealed that flavonoids may protect against cognitive decline in conditions such as Alzheimer’s disease, kidney disease, and chronic lower respiratory diseases, which are associated with a high mortality risk. Furthermore, our nomogram based on the risk score provided a feasible method to estimate the risk of mortality and guide the development of a tailored dietary plan focusing on specific dietary components and high-risk population subgroups.

The difference between our results and those from previous studies may be due to differences in the types of foods evaluated in questionnaires, varieties in dietary patterns of the cohorts, and differences in databases used to evaluate flavonoid intake. The content of dietary flavonoids may be influenced by many factors, including the diversity of plant phyla, order, family, species, and parts; conditions related to planting, such as soil type, regional climate conditions, and food maturity; and processing and cooking methods. It is also difficult to estimate flavonoid intake in the assessment of various mixed dishes. In addition to estimation errors, flavonoid metabolism varies widely among individuals (51). The biological activity of flavonoids is influenced by their absorption, bioavailability, distribution, metabolism, and elimination processes (28). Furthermore, gut microbes are known to alter the bioactivity, bioavailability, and toxicity of flavonoids (52). As shown in our results that a weak correlation between isoflavone intake and their corresponding urinary metabolites, we may infer that flavonoid intake only partially reflects the amounts of flavonoids exert biological effects in the body, which is a limitation of our study.

Our study had several limitations. Full multivariable Cox analyses should be employed to assess the significance of flavonoids in mortality. The misclassification of dietary exposure may be related to the fact that we only relied on the dietary intake from the questionnaire at baseline, and no information about changes in dietary intake during the follow-up. Additionally, the results of observational studies cannot infer causality. This should be investigated based on the specific single-disease mortality.

In summary, total consumption of flavonols, anthocyanidins, and isoflavones were significantly associated with a decreased risk of all-cause mortality. The risk scores obtained from the intake of survival-related flavonoids effectively evaluated the risk of death. The nomogram based on the risk score showed a good performance in estimating the possibility of death. From the perspective of public health, our findings provide novel insights for evaluating mortality risk based on flavonoid intake and establishing future personalized recommendations of flavonoid intake for good clinical outcomes. However, further well-designed and randomized controlled trials are needed to assess the health benefits of flavonoids.

## Data availability statement

Publicly available datasets were analyzed in this study. This data can be found at: https://www.cdc.gov/nchs/nhanes/about_nhanes.htm.

## Ethics statement

The studies involving human participants were reviewed and approved by the National Center for Health Statistics Research Ethics Review Board. The original study protocol is accessible on the website of the Ethics Review Board of the National Center for Health Statistics Research (https://www.cdc.gov/nchs/nhanes/irba98.htm), duly approved by the ethical review committee (protocol #2005–06; #2011–17). Our study was based on the public data from NHANES and all details are from the official website (https://www.cdc.gov/nchs/nhanes/about_nhanes.htm). The patients/participants provided their written informed consent to participate in this study.

## Author contributions

FZ and YZ analyzed the data and wrote the initial draft of the manuscript. YZ and KG designed the project and wrote the manuscript. YZ supervised the study. All authors contributed to the article and approved the submitted version.

## Funding

This project was supported by the Wuxi Taihu Lake Talent Plan, Supports for Leading Talents in Medical and Health Profession, Project Plan of Wuxi Institute of Translational Medicine (LCYJ202210), Scientific Research Project of Wuxi Commission of Health (M202041), Maternal and Child Health Research Project of Jiangsu Commission of Health (F202009), and Scientific Research Project of Jiangsu Maternal and Child Health Association (FYX202016).

## Conflict of interest

The authors declare that the research was conducted in the absence of any commercial or financial relationships that could be construed as a potential conflict of interest.

## Publisher’s note

All claims expressed in this article are solely those of the authors and do not necessarily represent those of their affiliated organizations, or those of the publisher, the editors and the reviewers. Any product that may be evaluated in this article, or claim that may be made by its manufacturer, is not guaranteed or endorsed by the publisher.
