# Supplementary material for: Flavonoid intake is associated with lower all-cause and disease-specific mortality: The National Health and Nutrition Examination Survey 2007–2010 and 2017–2018
Source: Front Nutr. 2023 Feb 14;10:1046998. doi: 10.3389/fnut.2023.1046998 (PMC9971007; doi:10.3389/fnut.2023.1046998)
Supplement: Supplementary file 1 [file Data_Sheet_1.docx]

Supplementary Material

# Supplementary Tables

Supplementary Table S1. The characteristic of the participants without flavonoid assessment

|  | n | | | Percentage (%) |
| --- | --- | --- | --- | --- |
| Race |  | | |  |
| White | 1437 | | | 0.35 |
| Black | 801 | | | 0.20 |
| Mexican | 770 | | | 0.19 |
| Other | 1040 | | | 0.26 |
| Sex |  | | |  |
| Female | 1931 | | | 0.48 |
| Male | 2117 | | | 0.52 |
| Education |  | | |  |
| 1 | 619 | | | 0.15 |
| 2 | 676 | | | 0.17 |
| 3 | 916 | | | 0.23 |
| 4 | 958 | | | 0.24 |
| 5 | 639 | | | 0.16 |
| Unknown | 240 | | | 0.06 |
| Marital status | |  | |  |
| 1 | | 1756 | | 0.43 |
| 2 | | 440 | | 0.11 |
| 3 | | 142 | | 0.04 |
| 4 | | 751 | | 0.19 |
| 5 | | 422 | | 0.10 |
| 6 | | 305 | | 0.08 |
| Unknown | | 232 | | 0.06 |
| Age, years |  | |  | |
| 18-39 | 1570 | | 0.3878 | |
| 40-59 | 1121 | | 0.2769 | |
| >=60 | 1357 | | 0.3352 | |

Supplementary Table S2**.** Study sample characteristics by living status data, NHANES 2007–2010, 2017–2018 until December 31, 2019

|  | **Alive, n= 12426** | **Deceased, n=1603** | | **P value** |
| --- | --- | --- | --- | --- |
| **Intake of flavonoids (mg/day)** | | | | |
| Daidzein | 0.82±0.06 | 0.42±0.12 | | 0.003 |
| Genistein | 1.16±0.08 | 0.58±0.16 | | 0.002 |
| Glycitein | 0.17±0.01 | 0.09±0.03 | | 0.01 |
| Cyanidin | 2.68±0.20 | 2.70±0.37 | | 0.96 |
| Petunidin | 1.21±0.10 | 1.00±0.19 | | 0.27 |
| Delphinidin | 1.75±0.14 | 1.26±0.21 | | 0.04 |
| Malvidin | 4.94±0.31 | 4.43±0.55 | | 0.38 |
| Pelargonidin | 1.64±0.12 | 1.62±0.30 | | 0.96 |
| Peonidin | 2.18±0.17 | 1.13±0.17 | | <0.0001 |
| Catechin | 7.85±0.18 | 7.35±0.40 | | 0.18 |
| Epigallocatechin | 16.83±0.84 | 15.12±1.36 | | 0.25 |
| Epicatechin | 10.19±0.25 | 9.14±0.49 | | 0.05 |
| Epicatechin 3-gallate | 10.83±0.55 | 9.99±0.94 | | 0.41 |
| Epigallocatechin 3-gallate | 29.08±1.73 | 25.19±2.06 | | 0.12 |
| Theaflavin | 1.58±0.09 | 1.65±0.20 | | 0.75 |
| Thearubigins | 90.48±4.63 | 92.55±10.47 | | 0.86 |
| Eriodictyol | 0.18±0.01 | 0.12±0.01 | | <0.0001 |
| Hesperetin | 8.70±0.24 | 11.10±0.82 | | 0.003 |
| Naringenin | 3.36±0.16 | 3.72±0.26 | | 0.11 |
| Apigenin | 0.24±0.03 | 0.17±0.01 | | 0.01 |
| Luteolin | 0.72±0.02 | 0.53±0.03 | | <0.0001 |
| Isorhamnetin | 0.87±0.02 | 0.71±0.04 | | <0.0001 |
| Kaempferol | 4.71±0.10 | 3.90±0.25 | | 0.002 |
| Myricetin | 1.55±0.04 | 1.36±0.08 | | 0.03 |
| Quercetin | 11.44±0.19 | 10.35±0.43 | | 0.01 |
| Theaflavin-3,3’-digallate | 1.74±0.10 | 1.83±0.23 | | 0.73 |
| Theaflavin-3’-gallate | 1.47±0.08 | 1.55±0.19 | | 0.75 |
| Theaflavin-3-gallate | 1.25±0.07 | 1.32±0.16 | | 0.7 |
| Gallocatechin | 1.71±0.08 | 1.66±0.17 | | 0.8 |
| Subtotal catechins | 76.49±3.49 | 68.45±5.26 | | 0.17 |
| Total isoflavones | 2.15±0.16 | 1.08±0.30 | | 0.002 |
| Total anthocyanidins | 14.39±0.75 | 12.15±1.59 | | 0.17 |
| Total flavan-3-ols | 173.01±7.49 | 167.34±16.22 | | 0.75 |
| Total flavanones | 12.23±0.37 | 14.94±1.02 | | 0.01 |
| Total flavones | 0.96±0.04 | 0.71±0.04 | | <0.0001 |
| Total flavonols | 18.58±0.32 | 16.31±0.75 | | 0.003 |
| Total sum of all 29 flavonoids | 221.32±7.73 | 212.52±17.67 | | 0.64 |
| **Baseline sociodemographic, lifestyle, and health-related variables** | | | | |
| Age, years | 45.43±0.30 | 66.85±0.57 | | < 0.0001 |
| Sex, % |  |  | | 0.02 |
| Female | 53.01(51.85,54.16) | 48.43(45.22,51.64) |  | |
| Male | 46.99(45.84,48.15) | 51.57(48.36,54.78) |  | |
| Race, % |  |  | <0.0001 | |
| Black | 11.42(9.60,13.24) | 11.47(9.10,13.83) |  | |
| Mexican | 9.05(7.01,11.09) | 3.52(1.70, 5.35) |  | |
| Other | 13.63(11.66,15.61) | 5.55(3.54, 7.57) |  | |
| White | 65.90(62.19,69.60) | 79.46(75.44,83.47) |  | |
| Education, % |  |  | <0.0001 | |
| 1 | 4.42(3.77, 5.07) | 11.92(8.90,14.95) |  | |
| 2 | 9.95(9.04,10.86) | 19.12(15.24,23.01) |  | |
| 3 | 24.82(23.28,26.36) | 26.03(23.38,28.68) |  | |
| 4 | 30.65(29.23,32.08) | 24.36(20.85,27.87) |  | |
| 5 | 30.16(27.51,32.80) | 18.56(15.04,22.09) |  | |
| Marital status, % |  |  | <0.0001 | |
| 1 | 55.75(53.68,57.82) | 47.81(43.35,52.26) |  | |
| 2 | 9.70(8.99,10.42) | 11.30(9.05,13.56) |  | |
| 3 | 2.51(2.12,2.91) | 1.99(0.98,3.00) |  | |
| 4 | 19.72(18.25,21.18) | 10.82(7.73,13.91) |  | |
| 5 | 4.21(3.71, 4.71) | 24.68(20.72,28.64) |  | |
| 6 | 8.10(7.16,9.04) | 3.41(2.10,4.71) |  | |
| PIR | 3.07±0.05 | 2.49±0.08 | <0.0001 | |
| BMI (kg/m^2^) | 29.11±0.14 | 29.50±0.30 | 0.27 | |
| Smoking status, % |  |  | < 0.0001 | |
| Former | 23.33(21.93,24.73) | 38.03(34.60,41.45) |  | |
| Never | 57.95(55.84,60.07) | 39.50(35.65,43.35) |  | |
| Now | 18.72(17.33,20.10) | 22.47(19.35,25.59) |  | |
| Alcohol usage, % |  |  | <0.0001 | |
| Former | 9.34(8.15,10.52) | 32.62(29.56,35.69) |  | |
| Heavy | 22.81(21.34,24.28) | 10.41(8.20,12.61) |  | |
| Mild | 38.74(36.81,40.66) | 32.54(27.85,37.23) |  | |
| Moderate | 18.88(17.78,19.98) | 8.43(6.58,10.28) |  | |
| Never | 10.24(9.29,11.18) | 16.00(13.05,18.96) |  | |
| Total score of HEI | 53.20±0.36 | 54.17±0.60 | 0.05 | |
| DII | 1.45±0.05 | 1.80±0.07 | <0.0001 | |
| Total time of PA (mins/week) | 1300.49±36.02 | 884.01±76.54 | <0.0001 | |
| Total MET of PA (/week) | 5201.98±144.10 | 3536.04±306.17 | <0.0001 | |
| **Disease diagnosis at interview** | | | | |
| Hyperlipidemia, % | | | <0.0001 | |
| No | 32.12(30.37,33.87) | 20.93(18.25,23.61) |  | |
| Yes | 67.88(66.13,69.63) | 79.07(76.39,81.75) |  | |
| Cardiovascular disease, % | | | <0.0001 | |
| No | 93.17(92.34,94.00) | 67.39(63.82,70.95) |  | |
| Yes | 6.83(6.00,7.66) | 32.61(29.05,36.18) |  | |
| Respiratory system disease, % | | | <0.0001 | |
| ACO | 1.86(1.50,2.22) | 5.82(4.55,7.08) |  | |
| Asthma | 12.04(11.06,13.02) | 8.08(5.92,10.24) |  | |
| COPD | 2.48(1.99,2.97) | 10.05(6.91,13.18) |  | |
| No | 83.63(82.31,84.94) | 76.06(72.43,79.68) |  | |
| Stroke, % | | | <0.0001 | |
| No | 97.49(97.13,97.85) | 87.08(83.84,90.33) |  | |
| Yes | 2.51(2.15,2.87) | 12.92(9.67,16.16) |  | |
| Cancer, % | | | <0.0001 | |
| No | 91.39(90.75,92.04) | 75.18(71.90,78.46) |  | |
| Yes | 8.61(7.96,9.25) | 24.82(21.54,28.10) |  | |
| Hypertension, % | | | <0.0001 | |
| No | 65.91(64.19,67.63) | 30.74(26.66,34.81) |  | |
| Yes | 34.09(32.37,35.81) | 69.26(65.19,73.34) |  | |
| DM, % | | | <0.0001 | |
| No | 80.85(79.56,82.14) | 55.69(51.25,60.13) |  | |
| Yes | 19.15(17.86,20.44) | 44.31(39.87,48.75) |  | |

Continuous variables are presented as mean ± standard deviation and categorical variables are presented as percentage (95% confidence intervals, 95% CIs). PIR, poverty income ratio; BMI, body mass index; HEI, Healthy Eating Index 2015 version; DII, dietary inflammatory index; PA, physical activity; MET, metabolic equivalent; COPD, chronic obstructive pulmonary disease; DM, type 2 diabetes mellitus. In education, less than 9th grade was coded as 1; 9–11th grade (includes 12th grade with no diploma) as 2, high school graduation or equivalent as 3, some college or associates degree as 4, and college graduate or above as 5. For marital status, married was coded as 1, divorced as 2, separated as 3, never married as 4, widowed as 5, and living with partner as 6.

Supplementary Table S3. The coefficients of each flavonoid by univariate Cox hazards regression analysis for all-cause mortality in the training set

| Variable | Estimate (coefficient) | HR | 95% CI | P value |
| --- | --- | --- | --- | --- |
| Eriodictyol (mg) | -0.33631225 | 0.71 | (0.62,0.83) | <0.0001 |
| Luteolin (mg) | -0.23193206 | 0.79 | (0.71,0.88) | <0.0001 |
| Total Flavones (mg) | -0.19419173 | 0.82 | (0.75,0.90) | <0.0001 |
| Isorhamnetin (mg) | -0.14306238 | 0.87 | (0.80,0.94) | <0.001 |
| Quercetin (mg) | -0.01562138 | 0.99 | (0.97,1.00) | 0.0035 |
| Total Flavonols (mg) | -0.01095984 | 0.99 | (0.98,1.00) | 0.0043 |
| Naringenin (mg) | 0.00628024 | 1.01 | (1.00,1.01) | 0.0056 |
| Kaempferol (mg) | -0.03645654 | 0.96 | (0.94,0.994) | 0.0144 |
| Apigenin (mg) | -0.2647471 | 0.77 | (0.61,0.97) | 0.0247 |
| Total Flavanones (mg) | 0.00259663 | 1.00 | (1.00,1.01) | 0.0376 |
| Peonidin (mg) | -0.01521517 | 0.999 | (0.97,1.00) | 0.0513 |
| Epicatechin (mg) | -0.00904074 | 0.99 | (0.98,1.00) | 0.0532 |
| Hesperetin (mg) | 0.00319489 | 1.00 | (1.00,1.01) | 0.0638 |
| Catechin (mg) | -0.00974735 | 0.99 | (0.98,1.00) | 0.0815 |
| Myricetin (mg) | -0.03874083 | 0.96 | (0.92,1.01) | 0.0853 |
| Epigallocatechin 3-gallate (mg) | -0.00140098 | 1.00 | (1.00,1.00) | 0.0883 |
| Subtotal Catechins (mg) | -0.00060018 | 1.00 | (1.00,1.00) | 0.1378 |
| Delphinidin (mg) | -0.01237627 | 0.99 | (0.97,1.01) | 0.1796 |
| Epigallocatechin (mg) | -0.00190181 | 1.00 | (1.00,1.00) | 0.2178 |
| Pelargonidin (mg) | -0.00934352 | 0.99 | (0.98,1.01) | 0.2200 |
| Epicatechin_3_gallate (mg) | -0.00260339 | 1.00 | (0.99,1.00) | 0.2756 |
| Total Anthocyanidins (mg) | -0.00170145 | 1.00 | (1.00,1.00) | 0.2837 |
| Daidzein (mg) | -0.04217708 | 0.96 | (0.89,1.04) | 0.2855 |
| Genistein (mg) | -0.0295318 | 0.97 | (0.92,1.03) | 0.2862 |
| Total Isoflavones (mg) | -0.01592615 | 0.98 | (0.96,1.01) | 0.2947 |
| Total Sum of all 29 flavonoids (mg) | -0.00010001 | 1.00 | (1.00,1.00) | 0.3696 |
| Glycitein (mg) | -0.16287193 | 0.85 | (0.58,1.25) | 0.4091 |
| Gallocatechin (mg) | -0.01409892 | 0.99 | (0.95,1.02) | 0.4307 |
| Total Flavan-3-ols (mg) | -0.00010001 | 1.00 | (1.00,1.00) | 0.4344 |
| Petunidin (mg) | -0.00722605 | 0.99 | (0.97,1.02) | 0.5561 |
| Thearubigins (mg) | -0.00010001 | 1.00 | (1.00,1.00) | 0.6652 |
| Theaflavin (mg) | -0.00440971 | 1.00 | (0.96,1.03) | 0.8086 |
| Theaflavin-3’-gallate (mg) | -0.00461061 | 1.00 | (0.96,1.03) | 0.8128 |
| Theaflavin-3,3’-digallate (mg) | -0.00380724 | 1.00 | (0.97,1.03) | 0.8164 |
| Theaflavin-3-gallate (mg) | -0.00501254 | 1.00 | (0.95,1.04) | 0.8242 |
| Malvidin (mg) | -0.00050013 | 1.00 | (0.99,1.01) | 0.8701 |
| Cyanidin (mg) | 9.9995E-05 | 1.00 | (1.00,1.01) | 0.9655 |

# Supplementary Figures


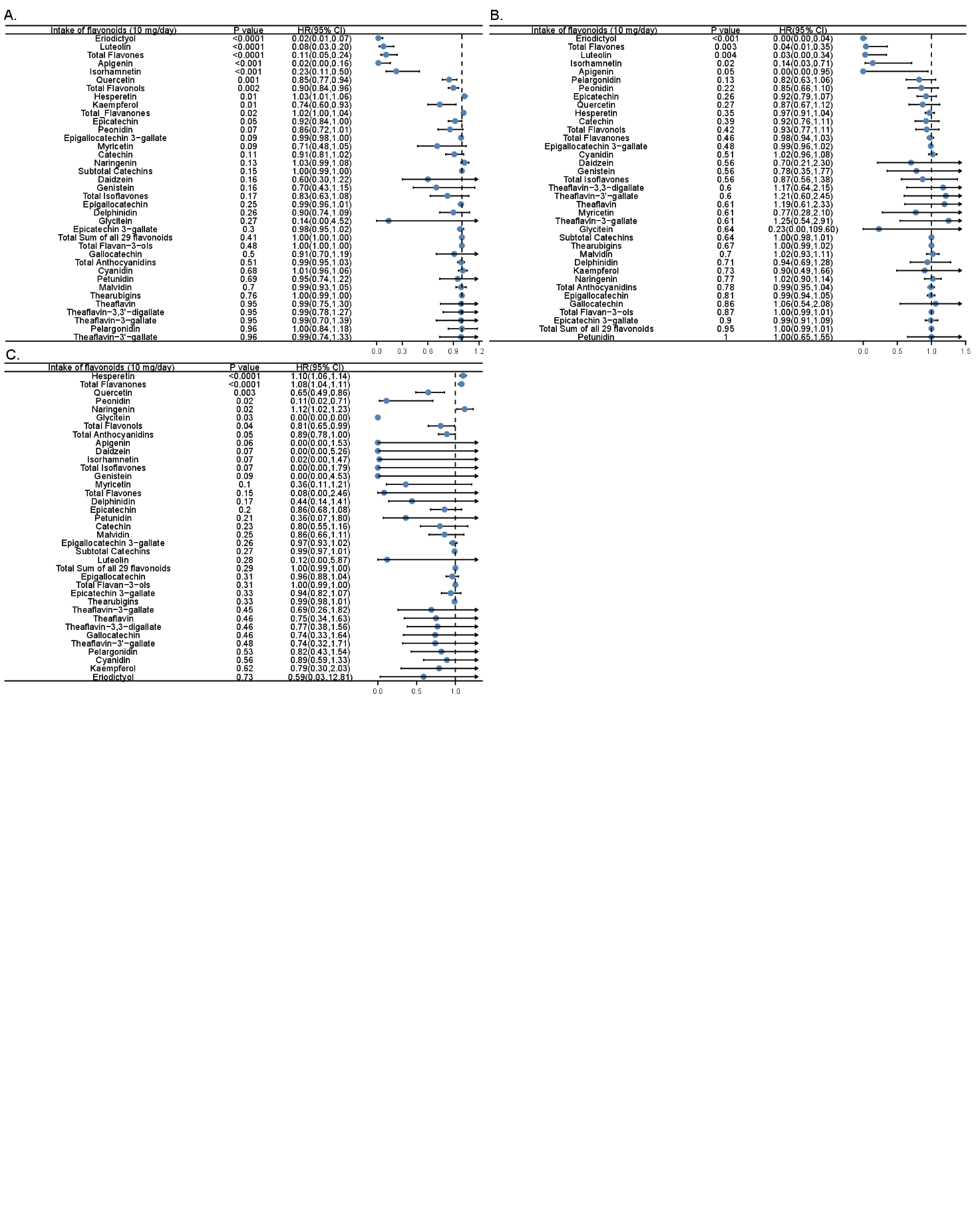


**Supplementary Figure S1.** Forest plots showing the association between flavonoid intake (mg*0.1/day) and all-cause mortality (A), mortality of heart diseases (B), and mortality of cerebrovascular diseases (C) in unadjusted Cox analysis with the increment unit of 10 mg/day. HR: Hazard ratios.


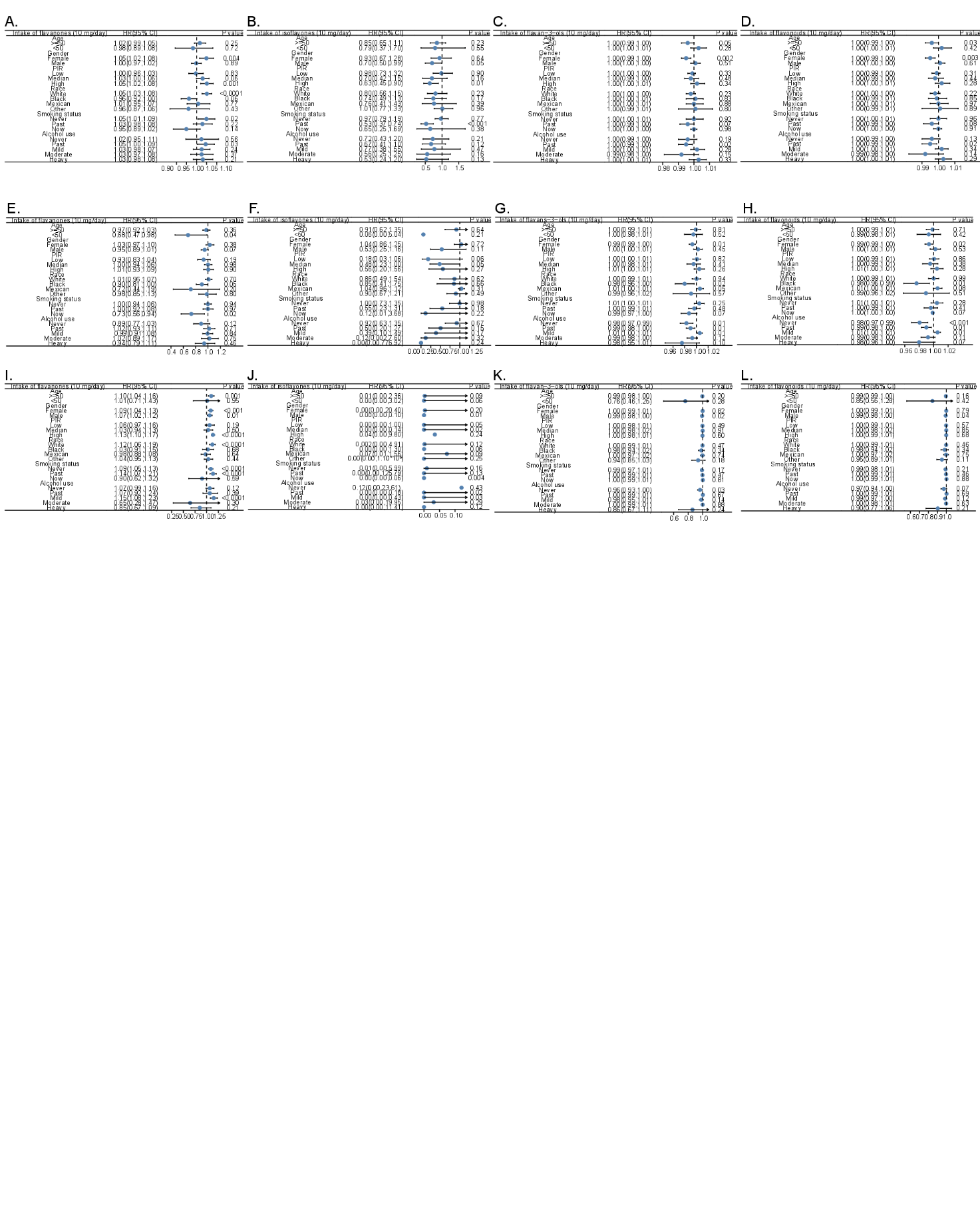


**Supplementary Figure S2.** Forest plots displaying the association between flavanone intake and all-cause mortality (A), mortality of heart diseases (E), and mortality of cerebrovascular diseases (I); the association between isoflavone intake and all-cause mortality (B), mortality of heart diseases (F), and mortality of cerebrovascular diseases (J); the association between flavan-3-ol intake and all-cause mortality (C), mortality of heart diseases (F), and mortality of cerebrovascular diseases (I); and the association between total 29 flavonoid intake and all-cause mortality (D), mortality of heart diseases (H), and mortality of cerebrovascular diseases (L) in unadjusted Cox analysis stratified by age, gender, PIR, race, smoking status, and alcohol usage with the increment unit of 10 mg/day. HR: Hazard ratios; PIR: poverty income ratio.


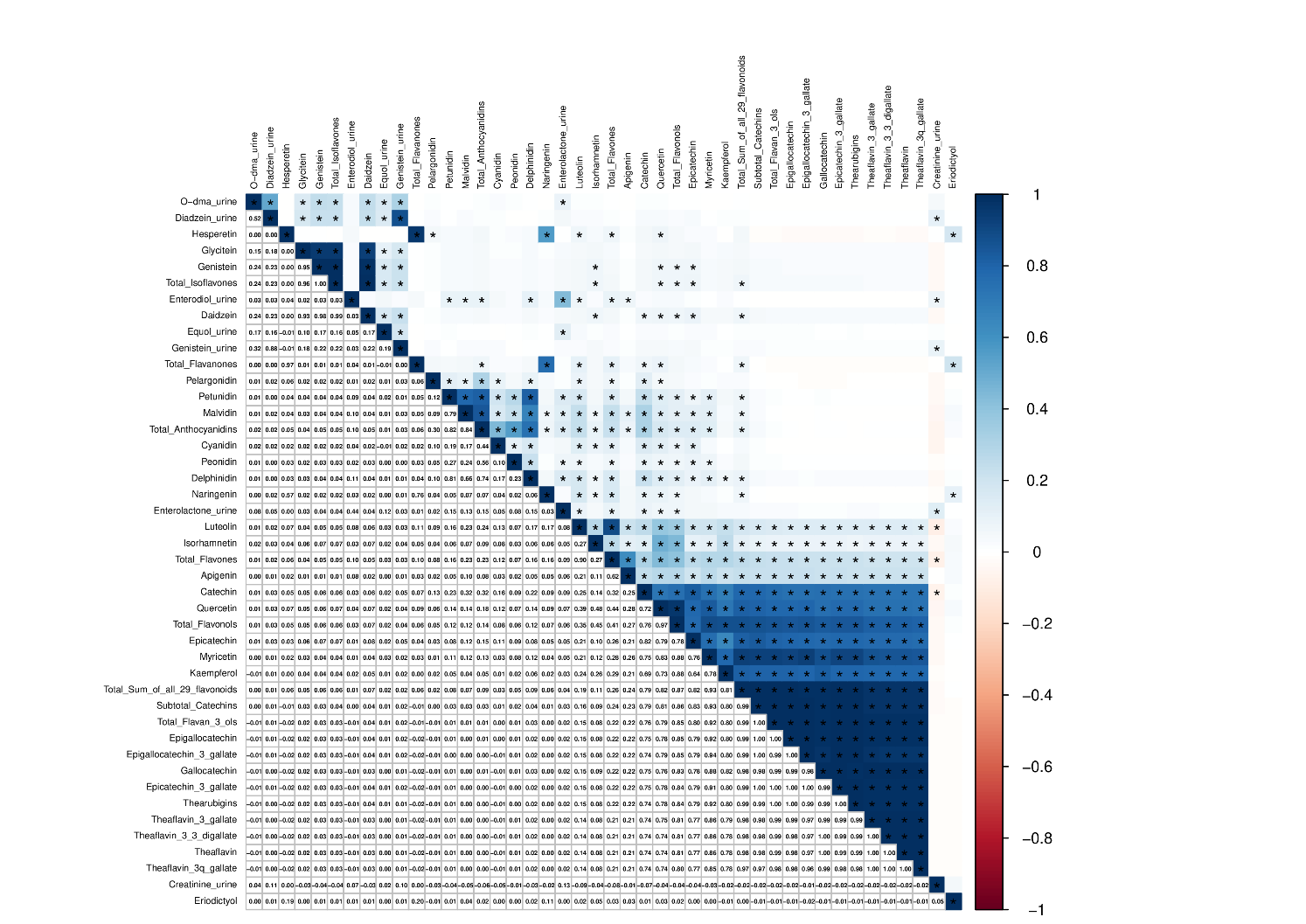


**Supplementary Figure S3.** Correlations between flavonoid intakes and urinary phytoestrogens, calculated by Pearson’s method. The numbers in the lower left half of the correlation graph represent the Pearson’s correlation coefficients. The color bars represent the Pearson’s correlation coefficients, dark blue as positive correlation and red as negative correlation. The asterisk * stands for P < 0.0001.
